# Spatiotemporal Variation in Avian Migration Phenology: Citizen Science Reveals Effects of Climate Change

**DOI:** 10.1371/journal.pone.0031662

**Published:** 2012-02-22

**Authors:** Allen H. Hurlbert, Zhongfei Liang

**Affiliations:** Department of Biology, University of North Carolina, Chapel Hill, North Carolina, United States of America; University of Osnabrueck, Germany

## Abstract

A growing number of studies have documented shifts in avian migratory phenology in response to climate change, and yet there is a large amount of unexplained variation in the magnitude of those responses across species and geographic regions. We use a database of citizen science bird observations to explore spatiotemporal variation in mean arrival dates across an unprecedented geographic extent for 18 common species in North America over the past decade, relating arrival dates to mean minimum spring temperature. Across all species and geographic locations, species shifted arrival dates 0.8 days earlier for every °C of warming of spring temperature, but it was common for some species in some locations to shift as much as 3–6 days earlier per °C. Species that advanced arrival dates the earliest in response to warming were those that migrate more slowly, short distance migrants, and species with broader climatic niches. These three variables explained 63% of the interspecific variation in phenological response. We also identify a latitudinal gradient in the average strength of phenological response, with species shifting arrival earlier at southern latitudes than northern latitudes for the same degree of warming. This observation is consistent with the idea that species must be more phenologically sensitive in less seasonal environments to maintain the same degree of precision in phenological timing.

## Introduction

The average surface air temperature on earth has warmed by approximately 0.74°C over the past century, with the most accelerated warming occurring over the past several decades [1]. In response to this large-scale warming, many organisms have shifted their distributions and altered the timing of seasonal life events such as flowering, growing, hatching, breeding and migrating [2]–[4]. Understanding how the strength and magnitude of such responses varies across species and with ecological context is critical for being able to predict the consequences of ongoing and future climate change and to identify species most at risk. Migration poses a particularly unique phenological challenge in that organisms must time their arrival with environmental conditions at distant locations. Individuals arriving too early may face adverse conditions and limited resources [5], while individuals arriving too late may face disadvantages in establishing breeding territories or finding high quality mates [6]–[8].

The extent to which migratory birds might alter the timing of migration in response to climate change depends in part upon the relative importance of endogenous versus exogenous controls. Migratory birds exhibit circannual rhythms of moult, gonad growth, migratory restlessness, and rapid fat accumulation that function to prepare the birds for migration and breeding [9]. These cycles may persist even in a controlled lab environment without seasonal cues, illustrating the endogenous component of migration timing [9], [10]. However, under natural conditions, birds also synchronize their migration to the seasons according to environmental cues such as photoperiod and temperature [10]. Photoperiod stays consistent year-to-year and is perhaps the most important time-keeper for migrants [5]. Other factors like weather conditions and temperature are much less predictable, and thus some measure of plasticity in migration timing is beneficial. In spring, temperature determines when food becomes available at certain latitudes, and migrants must be able to evaluate conditions en route and adjust their movements accordingly [5], [11], [12]. The ability to be flexible allows migrants to avoid frost and take advantage of resources as they become available, even in phenologically unusual years [5]. Thus, even if the onset of spring migration is primarily under endogenous control, arrival dates might still be expected to shift in response to changing climate.

In many regions, strong warming trends in average minimum temperature in early spring have led to an earlier start to the growing season [13], and concern has been raised over the ability of migrants to adjust to these changing environmental conditions [8], [14]. Recent work has attributed the earlier onset of arrival, breeding, and other life history events in a variety of migratory birds to rising temperatures in northern latitudes (e.g., [15], [16]). In a number of studies, the majority of species examined were shown to be arriving earlier in recent years or with warmer temperatures (e.g., [17]–[19]). However, there have also been studies in which few or none of the species examined displayed strong shifts in arrival date (e.g., [20]–[22]), with the majority of published studies lying between the two extremes (e.g., [23]–[27]). Clearly, the phenomenon of earlier spring arrival is far from universal, possibly due to the inconsistent warming trends around the world [13], [28] and to differences among species in ecology and life history traits (e.g., [29], [30]). Indeed, these sources of variation may ultimately provide the key to understanding the actual mechanisms underlying the relationship between migration timing and climate [31].

One of the most obvious differences among species that might influence the degree to which a species shifts arrival date is the average distance of migration. The endogenous circannual controls mentioned above appear to exert a stronger influence over long-distance migrants and limit their ability to adapt to important local weather signals compared to short-distance migrants ([32], [9], [33], but see [34]). In fact, some have argued that the inflexible migratory behavior of long distance migrants may be contributing to the decline of some species [8], [14], [35], [36]. In contrast, short-distance migrants, with a more flexible migration schedule, may be better able to assess local conditions and appear to have a more pronounced shift toward earlier migration (e.g., [19], [23], [37]–[39]). To date, this has been the most commonly examined species trait for explaining differences in phenological response to climate change in birds, and one that has received broad, although not universal, support [31].

Several other ecological and life history traits have been investigated to explain interspecific variation in phenological response to climate change as well. Végvári et al. [30] found that diet breadth was an important predictor, with species with broader diets exhibiting stronger phenological responses. In contrast, Møller et al. [36] found little evidence for an effect of habitat specialization. On the one hand, species that are generalists in terms of diet, habitat, or climatic niche may be less sensitive to phenological mismatches, and under weaker selection than specialists to respond adaptively. On the other hand, generalists might possess greater genetic variation or phenotypic plasticity making them more capable of exhibiting a phenological response. The effect of population size has also been explored in this context since it is expected to be positively related to genetic variation, but has received limited support so far [36], [40]. Several studies have identified a negative correlation between population trend and the degree to which a species has exhibited a phenological response to climate change [14], [36], although the presumed causality is typically that the ability to adaptively shift migratory phenology affects a species' population trend.

Studies of arrival date of migratory birds are often conducted using banding data from individual reserves and research stations at only one or a few locations. Few studies have examined the arrival of migratory birds over broader spatial extents (but see [41], [42]). The potential for such broad scale analyses of distribution and phenology has increased recently with the increase in public interest in conservation and the development of several online programs where amateur birders can submit their bird observations for science. The Cornell Laboratory of Ornithology and the National Audubon Society together organize one such program, eBird (http://www.ebird.org). Since its inception in November 2002, eBird has collected and compiled more than 48 million bird observations by over 35,000 contributors [43], [44]. In addition to new checklists, users have also entered historical observations, so that eBird includes data prior to 2002 as well. This wealth of data has great value in ecological research and conservation studies, and tapping into this resource has the potential to unveil novel spatiotemporal patterns (e.g., [45]). Although the relative novelty of the eBird program precludes the examination of multi-decadal time-series, its strength lies in the simultaneous characterization of arrival date over an unprecedented geographic breadth.

Here, we describe a novel and statistically robust method for characterizing arrival date from citizen science data, and then apply it to a spatially comprehensive dataset from eBird to investigate changes in arrival date of migratory birds between 2000 and 2010 across the eastern United States. This temporal window, although short, is of interest because of the year-to-year climatic variability that occurred in the absence of any strong regional trends [46]. We illustrate the variability in phenological shifts both across species and across geographic regions, and evaluate whether changes in the timing of migration can be attributed to corresponding changes in average spring temperature. We examine a variety of ecological traits that have been suggested elsewhere to influence the strength of a species' phenological response to changes in climate, and present a novel framework that predicts greater phenological sensitivity in less seasonal environments, consistent with our findings.

## Results

**Figure 1:**
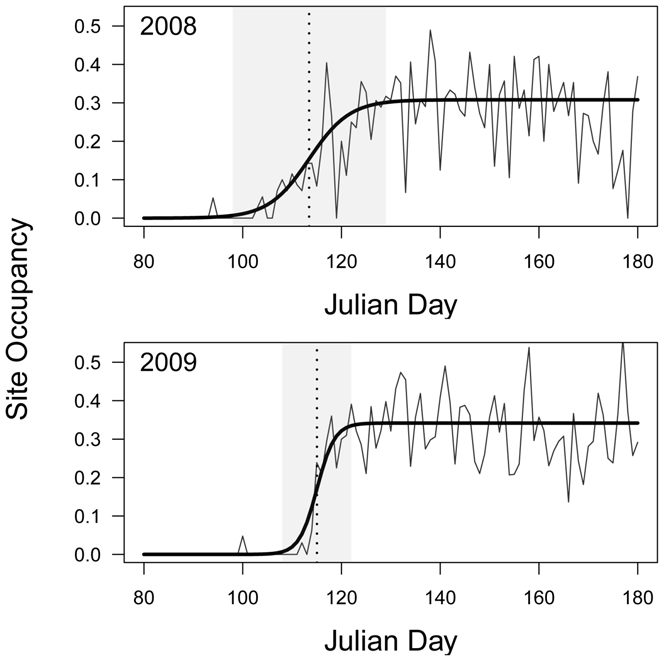
Estimating arrival date from temporal occupancy patterns. Proportion of checklist locations at which the House Wren (*Troglodytes aedon*) was observed from Julian days 80–180 (roughly 10 March to 30 June) within a 2-degree lat-long block centered at 41°N and 73°W in 2008 and 2009. Heavy line is the best fitting logistic curve to the data, and the vertical dotted line indicates the inflection point of that curve which is used as an estimate of mean arrival date. The shaded area indicates the region in which occupancy is between 2.5% and 97.5% of the asymptotic value, and the width of this area was used as a confidence interval on the arrival date estimate for weighting purposes.

We estimated a population-level arrival date for 18 common migratory species (Table 1) in 2° blocks for each year by fitting a logistic model to the proportion of unique checklist locations within the block where the focal species was observed as a function of Julian day (Figure 1). As individuals of a species arrive in a region, the proportion of sites at which they are observed should rise from zero to the value which represents the species' overall prevalence during the breeding season. We used the inflection point of the logistic fit as our measure of mean arrival date (MAD).

**Table 1:** The species analyzed in this study, along with species abbreviations, migration class, foraging guild, number of lat-long blocks analysed, and the median slope of mean arrival date (MAD) as a function of minimum spring temperature over all lat-long blocks.

Species	Common Name	Code	Migration distance^1^	Foraging guild^2^	*n*	MAD shift per °C
*Myiarchus crinitus*	Great crested flycatcher	GCFL	Long	A	23	−1.20
*Vireo olivaceus*	Red-eyed vireo	REVI	Long	F	26	−1.13
*Geothlypis trichas*	Common yellowthroat	COYE	Short	F	25	−1.04
*Troglodytes aedon*	House wren	HOWR	Short	F	23	−0.98
*Passerina cyanea*	Indigo bunting	INBU	Long	F	25	−0.97
*Piranga olivacea*	Scarlet tanager	SCTA	Long	F	17	−0.86
*Chaetura pelagica*	Chimney swift	CHSW	Long	A	21	−0.86
*Mniotilta varia*	Black-and-white warbler	BAWW	Short	F	9	−0.85
*Tyrannus tyrannus*	Eastern kingbird	EAKI	Long	A	16	−0.75
*Dendroica petechia*	Yellow warbler	YEWA	Long	F	27	−0.68
*Seiurus aurocapilla*	Ovenbird	OVEN	Short	G	17	−0.66
*Icterus galbula*	Baltimore oriole	BAOR	Short	F	28	−0.55
*Hylocichla mustelina*	Wood thrush	WOTH	Long	G	23	−0.47
*Catharus fuscescens*	Veery	VEER	Long	G	10	−0.45
*Setophaga ruticilla*	American redstart	AMRE	Long	F	15	−0.37
*Hirundo rustica*	Barn swallow	BARS	Long	A	15	−0.27
*Contopus virens*	Eastern wood-pewee	EAWP	Long	A	15	0.05
*Pheucticus ludovicianus*	Rose-breasted grosbeak	RBGR	Long	F	24	0.35

1 Technically, all of these species may be considered Neotropical migrants, but here we define those that winter at least partially in the U.S. as short distance migrants.

2 A - aerial insectivore, F - foliage gleaner, G - ground gleaner.

**Figure 2:**
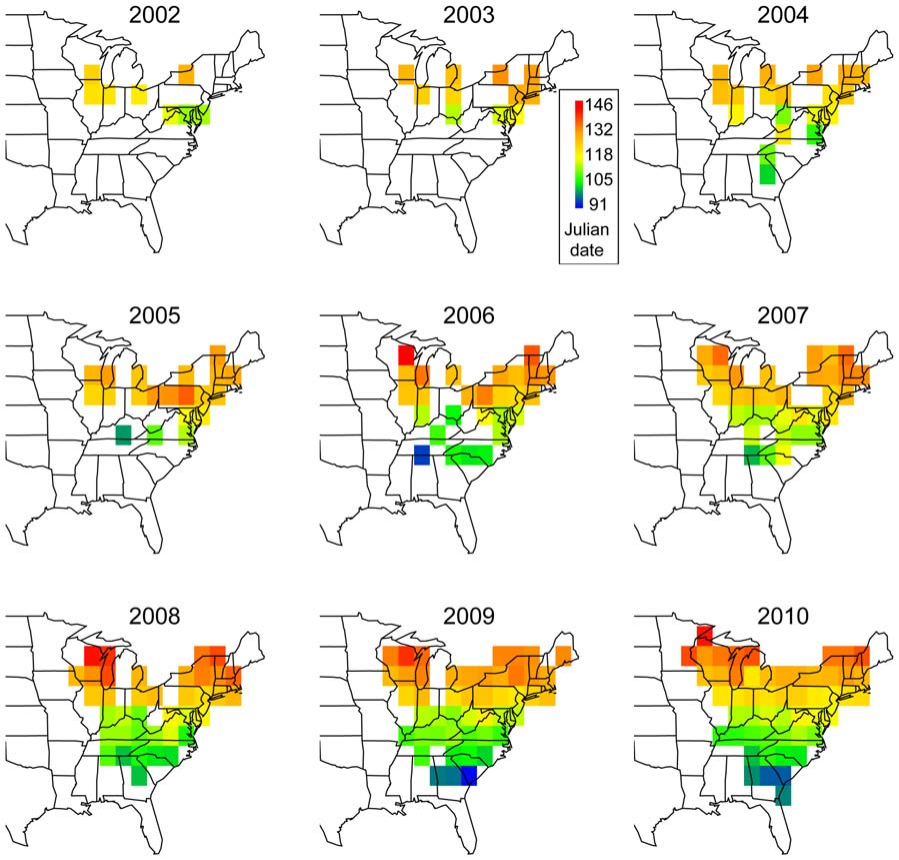
Spring arrival dates for the red-eyed vireo. Spring arrival dates estimated from citizen science data collection efforts for the red-eyed vireo (*Vireo olivaceus*) across 2° lat-long-blocks in eastern North America from 2002–2010.

Although the spatial coverage of eBird data is presently quite thorough across eastern North America, gaps in coverage become increasingly apparent going back in time (Figure 2). Nevertheless, these data allowed us to examine spatiotemporal variation in median arrival date across those regions with sufficient sampling effort. The red-eyed vireo (*Vireo olivaceus*) is one of the most common long distance migrants of eastern North America and therefore has the most geographically complete coverage in the dataset. Like all species examined (see all maps in Appendix S1), the red-eyed vireo demonstrates a strong latitudinal gradient in arrival date. In 2010, birds first arrived in Georgia in the first week of April, but did not arrive in the northeastern and north-central U.S. until mid- to late May (Figure 2).

**Figure 3:**
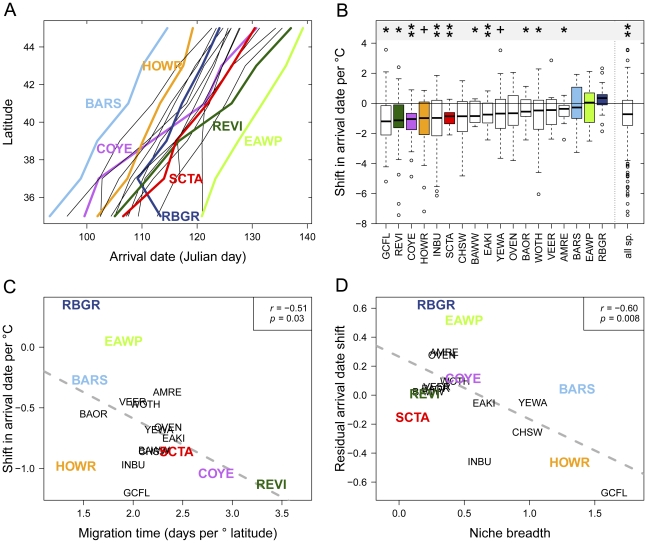
Explaining interspecific variation in phenological response. (A) Mean arrival date (averaged over both year and longitude) as a function of latitude for 18 bird species, depicting the rate at which various species advance northward during migration. (B) Boxplots showing the variation in the slope of the trend in arrival date with minimum spring temperature for each species, with more negative values reflecting earlier arrival. +, p<0.10; *, p<0.05; **, p<0.01. (C) Relationship between migration time (from (A)) and the median phenological response of arrival date to temperature. (D) Residuals of the phenological response to temperature after controlling for migration time and migration distance as a function of niche breadth. Species codes are given in Table 1.

While all species exhibited the expected latitudinal gradient in arrival date, species differed in the average speed with which they advanced northward (Figure 3A). Red-eyed vireo and common yellowthroat (*Geothlypis trichas*) were among the species that advanced mostly slowly, taking 31–32 days to cover 10 degrees of latitude. In contrast, the house wren (*Troglodytes aedon*) and barn swallow (*Hirundo rustica*) covered the same distance in only 17–21 days on average. Thus, while the house wren and common yellowthroat tended to arrive in Georgia and South Carolina at roughly the same time each spring, the latter took an additional two weeks to arrive in New England and the Great Lakes region. Also evident in Figure 3A is the tendency for some species to be characteristically early or later arrivals. For example, while the eastern wood pewee (*Contopus virens*) migrated northward at speeds similar to the house wren and barn swallow, it was one of the latest species to arrive at any latitude while the latter two were among the earliest. Similarly, while the red-eyed vireo and common yellowthroat advanced northwards at similarly slow rates, the common yellowthroat tended to precede the vireo by 5–6 days at all latitudes.

Across all species and locations migrant MADs varied strongly with minimum spring temperature (0.80 days earlier per °C, *t* = −8.14, *p*<6.6e-16), although species differed in the strength of their responses (Figure 3B). The single strongest univariate predictor of the median MAD-temperature slope (referred to hereafter as the measure of phenological response) was migration time. Species that advanced northward more slowly during migration had stronger phenological responses to minimum spring temperature (Figure 3C, *R* ^2^ = 0.26, *p* = 0.03). One notable exception was the house wren, which is the species traveling the shortest distance on average from its wintering grounds of those considered here. Indeed, the most highly supported model included migration time, the categorical migration distance, and niche breadth as the best predictors of phenological response (for the next best model, ΔAIC_c_ = 2.66; Table 2). Species with broader climatic niches exhibited a stronger phenological response to temperature than expected once migration time and distance are taken into account (Figure 3D).

**Table 2:** Most supported models explaining interspecific variation in shifts in arrival date in response to temperature change.

	Top 5 Models					Variable weight
Model	1	2	3	4	5	na
R^2^	0.63	0.49	0.65	0.63	0.58	na
AIC_c_	12.86	15.52	16.02	16.76	17.07	na
ΔAIC_c_	0	2.66	3.16	3.90	4.21	na
w_i_	0.43	0.11	0.09	0.06	0.05	na
Migration speed	1	1	1	1	1	0.97
Niche breadth	1	1	1	1	1	0.92
Migration distance	1	0	1	1	0	0.68
Population trend	0	0	1	0	0	0.20
Foraging guild	0	0	0	1	0	0.14
Relative abundance	0	0	0	0	1	0.14

Top 5 models out of 63 as ranked by AIC_c_, including model weights and relative importance weights of each of the 6 variables considered. The variable importance weights represent the sum of the model weights for all models in which a particular variable is entered (Burnham and Anderson 2002). na, not applicable.

**Figure 4:**
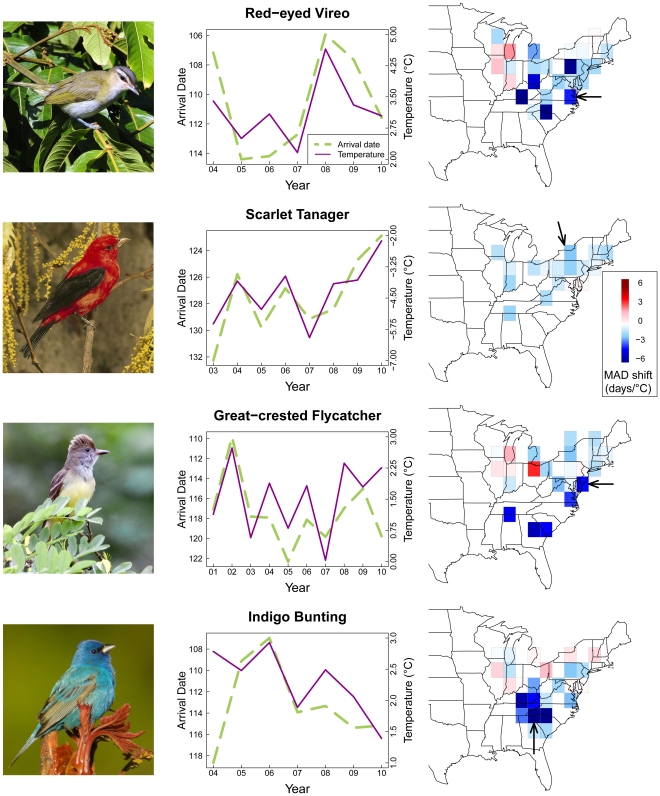
Geographic variation in phenological response and example trajectories of temperature and arrival date. Geographic variation in the mean shift in arrival date per °C change in minimum spring temperature for four bird species (right-hand column). The left-hand column depicts changes in both minimum spring temperature (solid line) and arrival date (dashed line) through time for one example region (indicated by arrow) for each species. Note that the arrival date axis increases towards the bottom. Photo credits: red-eyed vireo, Dario Sanches; scarlet tanager, Steve Maslowski; great-crested flycatcher, Matt Ward; indigo bunting, Kevin Bolton.

**Figure 5:**
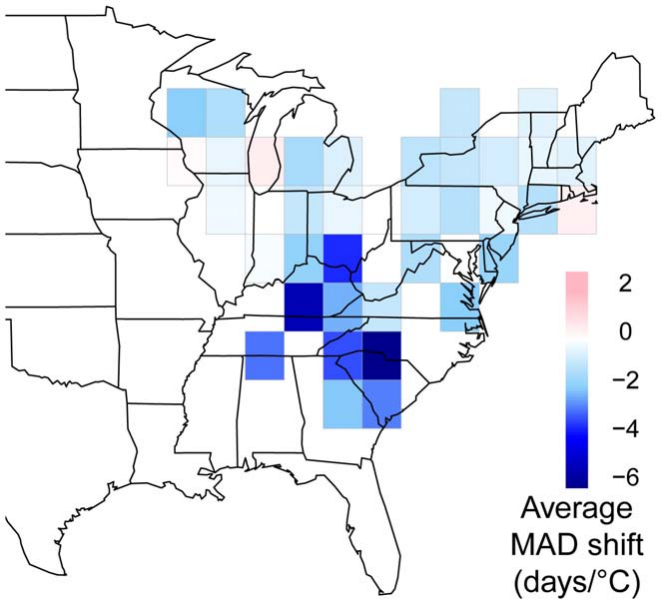
Geographical variation in the community-level phenological response. Mean shift in arrival date per °C change in minimum spring temperature for all grid cells with at least two species trends.

For all species, the rate of change of MAD through time varied heterogeneously across the breeding range, with species exhibiting a trend toward both earlier or later arrival over the past decade depending upon the region. However, much of this variation was consistent with a shift toward earlier arrival in years with warmer spring temperatures (Figure 4, blue cells). For a few species such as the scarlet tanager (*Piranga olivacea*), the manner in which MAD varied with spring temperature was fairly consistent across the range, while most species exhibited substantial geographic variation in temperature-MAD slopes (Figure 4). In every lat-long block with two or more species' time series, we calculated the mean temperature-MAD slope across species, and geographic variation in those values is shown in Figure 5. Caution is warranted in interpreting this pattern because species composition (true composition, as well as composition based on which species had sufficient data for analyses) varies across the grid, and thus differences may be due to differences in geography and climate or to compositional differences. Regardless, it appears that of the regions with sufficient data for analysis, species in the southeastern United States are shifting arrival dates earlier per degree Celsius than are species at more northern latitudes (*p* = 0.0002; Figure 5). This finding appears to hold intraspecifically as well, at least for some species (e.g., great crested flycatcher (*Myiarchus crinitus*), indigo bunting (*Passerina cyanea*), Figure 4).

## Discussion

Here we present the most spatially comprehensive study of migration phenology of North American birds using a large and growing database of citizen science observations. In agreement with a number of other studies conducted over longer time series but over just a few select locations (see reviews in [31], [40], [47]), we find that most species appear to time their arrival on breeding grounds based on climate or related factors. The fact that such relationships are apparent despite time series of 10 years or less is a testament to both the variability in minimum spring temperatures over this past decade and the magnitude of its effect on mean arrival dates. Furthermore, we found that arrival date is more closely related to spring temperature than year per se (unpublished analyses) in spite of the steady increase in the number of eBird observations through time, highlighting the fact that our method of estimating arrival dates is robust to variation in sampling effort. The greatest novelty of this study, however, is in its exploration of the variability in phenological responses to climate change across species and across space. We found that the single most important predictor of the strength of phenological response to temperature was the speed with which a species appeared to migrate northward. Species that advance more slowly may be better able to assess local conditions en route and may be better able to time their arrival with favorable conditions on the breeding ground. This is effectively the argument that has been made for why short distance migrants might respond more strongly than long distance migrants [19], [23], [37]–[39]. While we also found support for our binary migration distance variable, it was clearly of secondary importance to migration speed (relative importance weights [48] for the two variables 0.68 and 0.97, respectively).

One reason that migration speed *per se* has not received attention in the literature is that good, comparative estimates based on more than a handful of geographical locations have been lacking. Even when such estimates have been made, the focus has often been on explaining interannual variability (e.g., migration was faster in warm years, or phenologically early years) rather than on explaining interspecific differences (e.g., [28]). Our results suggest, however, that migration speed may be a critical trait that determines the vulnerability of species to climate change. Of course, the measure we use in this study is still rather coarse in that it is based on a species-level aggregate measure and at least one step removed from individual behavior. Measuring the rate of advancement of the overall migration front glosses over important migratory strategies of individual populations that may be leapfrogging each other rather than all proceeding north at equal rates in some treadmill fashion [5], [49]. Even comparisons of relative movement rates across species depend on individuals of all species following the same migratory pattern, either with first arrivals at a location being local breeders or first arrivals being transient birds moving further north. The extent to which the species analyzed here fall along this spectrum of migratory behavior is not well known, and more detailed insight into particular species will continue to be gained through studies that employ banding, satellite transmitters and geolocators (e.g., [50], [51]). Nevertheless, we expect that, at worst, differences between species in this aspect of migratory behavior introduce noise into our estimates of the rate of advancement of the overall migration front. Although our interpretation must be less precise than we would like, this coarse aggregate measure of migration speed appears to be capturing some important aspect of migration biology and makes it possible to compare across a large number of species for which no detailed studies have been conducted.

Of the other species-level variables examined, only climatic niche breadth was important for predicting the strength of phenological response to climate change. This measure of niche breadth is based on a multivariate consideration of the climate space occupied by each species [52], and is positively correlated with the strength of phenological response. This result is contrary to the expectation if generalists were to experience weaker selection than specialists with respect to compensatory shifts in arrival date. Rather, it supports the idea that generalist species are better able to respond phenologically to climate change either because they possess greater genetic variation, or greater phenotypic plasticity [53], [54]. In this study, the demonstrated phenological responses to variable, often non-trending changes in temperature over short time series (cf. Figure 4) suggest a greater role for plasticity than evolution in explaining the patterns reported here. Distinguishing between these two possibilities more generally has been identified as an important direction for future research [31].

We found a weak negative correlation between population trend over the past 44 years and the MAD shift per °C (*r* = −0.40, *p* = 0.10), consistent with several other recent studies [14], [36]. Three of the four species exhibiting positive trends in abundance―red-eyed vireo, great crested flycatcher, and house wren―also exhibit among the top four largest shifts in arrival date in response to temperature. This correlation is one that might be expected based purely on a sampling bias if arrival date were estimated based on the arrival date of the first individual [55], [56] as has been done in many previous studies. However, our methodology of fitting a logistic curve to occupancy data through time allows us to estimate arrival date (the location of the inflection point) independently of the asymptotic level of occupancy within the region (which could potentially be affected by regional abundance). Møller et al. [36] found a strong negative relationship between population trend and phenological response to climate change in European birds, arguing that the species that failed to exhibit a phenological response to climate change were more likely to decline in abundance. This perspective on cause and effect is supported by the observation that individuals that mistime their arrival on breeding grounds relative to the peak in resource availability tend to incur fitness costs in terms of the number and weight of fledglings produced [57]. Of particular concern for conservation is the potential for a positive feedback, in that a decrease in population size may reduce total genetic variation and hence constrain the ability of a species to respond adaptively to climate change, further hastening its decline [36], [58].

**Figure 6:**
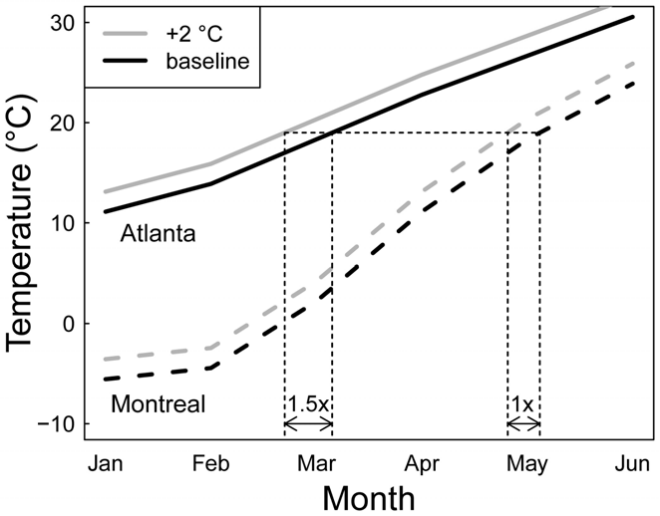
A model of increased temperature sensitivity in low seasonality environments. Seasonal variation in average monthly temperatures from January to June in Atlanta (solid) and Montreal (dashed) based on long term averages from weather.com (black) and under a seasonally uniform warming scenario of +2°C (gray). Dotted lines indicate the predicted arrival dates of a hypothetical species that based its arrival on an average temperature of 19°C under each of the four scenarios. The same degree of warming would result in a greater shift in arrival date in Atlanta.

Given that the earth's climate has been changing heterogeneously across the globe, it is not surprising that temporal trends in arrival date have been observed to vary spatially in the few studies that have put together geographically extensive datasets [41], [42]. We show here that heterogeneous climate change alone cannot explain this spatial variation in arrival date, however, as the average number of days by which species shift arrival date per °C also varies spatially. Specifically, a given change in spring temperature results in less of a shift at higher compared to lower latitudes within eastern North America (the mean MAD shift is ∼4 times earlier at 34°N compared to 42°N per °C). Rubolini et al. [41] identified a similar latitudinal trend in Europe. The weaker phenological response of MAD to temperature change at high latitudes occurs despite the fact that higher latitudes have experienced greater warming than lower latitudes over the past decade (Figure S1). This may reflect the fact that species using temperature cues in less seasonal environments may need to be more sensitive to those cues compared to species in more seasonal environments to maintain the same degree of precision in phenological timing. Because the rate of increase in temperature through the spring is faster at higher latitudes (50% faster in Montreal compared to Atlanta), a given temperature shift corresponds to a greater passage of time at lower latitudes (Figure 6). Under a scenario of seasonally uniform warming, an individual that based its spring arrival on a particular temperature would have to shift its arrival 50% earlier in Atlanta than it would in Montreal to achieve “compensatory advancement” as detailed by Saino et al. [59]. Geographic variation in the strength of phenological response to interannual variation in spring temperature appears to be consistent with variation in the steepness of the intraseasonal temperature gradient. Of course, this relationship may be complicated by the fact that temperatures are not warming uniformly across the seasons, and so this observation clearly merits further research.

Projections for climate change in North America suggest a continuation of the observed trend for greater warming at higher compared to lower latitudes. A suite of climate models and boundary conditions suggest that the northeastern U.S. is expected to warm 1.5–2.5°C by the period 2041–2070, while the southeastern U.S. is expected to warm 1–2°C [60]. For those species, such as the scarlet tanager (Figure 4C), that exhibit a geographically uniform response of MAD to temperature, arrival dates would therefore be expected to shift slightly earlier in the north compared to the south. However, for species such as the indigo bunting and great crested flycatcher, the difference in the magnitude of phenological response between south and north greatly exceeds the projected differential in warming. Individuals of these species are predicted to shift their arrival in the southeast by a week earlier or more, while in the northeast they are predicted to shift by at most 2–3 days. This implies that for those species that have demonstrated the ability to shift arrival dates in response to climate, that the slope of the latitudinal gradient in arrival date (cf. Figure 3A) may become shallower and that birds may end up spending longer in transit at migratory stopover sites. A critical assumption of these predictions, however, is that the observed shifts in arrival date are adaptive and well-timed relative to the proximate factors most important for survival and reproduction. This has not been the case for several species investigated in Europe [57], [61], and may mean that species must either begin shifting arrival dates more adaptively or face population decline [36].

While the eBird dataset we have employed has limitations―relatively short time series to date, variability in sampling effort and observer abilities―the sheer volume of observations (>3 million in May 2011 alone) presents an unparalleled opportunity for examining spatiotemporal distributions of avian species in North America (e.g., [45]). eBird also highlights the extraordinary potential for citizen science initiatives to generate novel datasets that can inform some of the most critical questions in the ecology, conservation biology, and global change arenas. While many studies have documented a response of arrival date to climate at select locations, our results highlight the geographic and taxonomic variability in response to climate change. We find that the species that are least able to adjust their migratory phenology are those that advance northward the fastest, and thus have perhaps the most inflexible migratory behavior. Furthermore, we find a latitudinal gradient in average phenological response that is steeper than the projected latitudinal gradient in temperature change, implying that many species will either significantly alter the rate and timing of migration or face phenological mismatches. The continued collection of citizen science data, in combination with datasets on plant and arthropod phenology, will yield even more powerful tests of these ideas and the ways in which a changing climate will impact bird communities across the globe.

## Materials and Methods

### Bird Data

We examined how spring arrival dates varied in space and through time for 18 common migratory birds of eastern North America with sufficient spatial coverage and data availability (Table 1). For the purposes of this study, any species with a substantial winter population within the continental U.S. was labeled a short distance migrant, and all others were labeled long distance migrants. Some short-distance migrants that overwinter throughout a large fraction of the study region were not examined due to the difficulty in distinguishing between overwintering individuals and newly arrived migrants. We assigned each species to one of three foraging guilds―aerial insectivore, foliage gleaner, or ground gleaner―based on assignments in Ehrlich et al. [62] and Birds of North America species accounts [63]. We also collated data on climatic niche breadth [52], and average relative abundance and population trend from 1966–2009 throughout eastern North America based on the Breeding Bird Survey [64].

We downloaded all reported observations from March through June of these 18 species for the years 2000–2010 from the online citizen science program eBird (downloaded 14 December 2010) for the United States east of the Mississippi River and for two Canadian provinces, Ontario and Quebec. Participants submit counts of bird species seen or heard in surveys of variable length and areal coverage from reported lat-long locations. Only complete checklists were used; casual single species observations and observations associated with Project Feeder Watch were discarded. In the instances where multiple checklists were conducted at the exact same location on the same day, we included only the checklist that recorded the highest number of individuals. These geographically referenced occurrence data were used to estimate arrival dates for each species within 2° lat-long blocks (see below).

While eBird observations are submitted throughout the year, participants are especially active during spring and fall migration making the dataset particularly suitable for examining questions of migration timing. Nevertheless, several caveats are best kept in mind when utilizing eBird data. First, probability of detection for any given species is expected to vary with observer ability as well as the duration and spatial coverage of the survey. We assume that variation in these determinants of survey quality is independent of date, year, geographic location, and annual temperature, and that this variation is primarily a source of noise. Second, the distribution of observer effort is non-random in both space and time. This limits both the spatial resolution with which we can examine patterns as well as the geographic regions and years in which arrival dates can reliably be estimated. In some regions, such as the extreme southeastern U.S., arrival date can only be estimated for recent years, while in parts of the Northeast and northern Midwest longer time series are available. This may lead to geographic variation in the error associated with effect sizes, but should not lead to any bias in the estimates themselves.

### Temperature Data

Historical temperature data at 4 km resolution were downloaded from the PRISM Climate Group (available online at http://www.prism.oregonstate.edu/). For each 2° block we calculated mean minimum daily temperatures averaged over February, March and April for each year. We also conducted analyses using mean maximum and average daily temperatures averaged over this period. As these measures are all positively correlated, results were qualitatively similar, and only results based on mean minimum temperature are reported.

### Analysis

The estimation of arrival dates is fraught with potential biases and analytical challenges. The most commonly studied metric of arrival is the first arrival date, indicated by the first individual of a species recorded in a migration season. However, this measure of arrival date is prone to the effects of outliers and fluctuations in sampling effort and population size, and therefore not necessarily descriptive of the migration behavior of the population as a whole [65], [66]. We instead utilized all of the species occurrence data in each year between Julian days 80 and 180 (from roughly 20 March to 30 June) to estimate a population-level arrival date of a species in 2° blocks. This was done by fitting a logistic model to the proportion of unique checklist locations within the block where the focal species was observed as a function of Julian day (Figure 1) (see [67] for a similar approach using a fourth-order polynomial). The proportion of sites at which a focal species is observed is expected to rise asymptotically from zero in the winter to the value which represents the species' overall prevalence during the breeding season. Note, however, that we are unable to distinguish between individuals that have arrived to breed in the area versus those passing through. The use of a proportion of sites rather than the absolute number of sites or the absolute number of birds makes the measure more robust to daily variation in observation effort, and the use of unique sites rather than unique checklists reduces the probability of double-counting birds that may have been seen by different observers at the same location. We used the inflection point of the logistic fit as our measure of mean arrival date (MAD). In addition, we calculated a confidence interval on the estimated MAD based on the range of days over which the probability of occupancy was between 2.5% and 97.5% of the asymptotic value (Figure 1).

We note that an underlying assumption of our analysis is that the logistic curve is a universally appropriate approximation of how occupancy changes over the course of spring migration. In fact, there are reasons to believe that occupancy might actually decline after reaching some initial peak due to the reduced detectability of singing birds as they initiate nesting [67], or to the passage of an initial wave of migrants that might be observed in a broader range of habitats than local breeders. To evaluate this possibility, for all time series we systematically re-fit a logistic curve to temporal windows that extended to varying lengths beyond the previously estimated inflection point. We then examined the relationship between window length and newly estimated inflection point location. The asymptote tended to decrease with longer windows in 67% of time series, resulting in marginally earlier inflection points. However, there was no latitudinal signature in where this effect was strongest (*p* = 0.84), and there was also no difference between short distance migrants and long distance migrants in the tendency for temporal window length to affect arrival date estimates (*p* = 0.21). These analyses suggest that a sigmoid curve adequately describes the vast majority of arrival patterns, and also indicate that any exceptions did not cause artifacts either in the latitudinal patterns that we describe or artifacts in comparisons of short- and long-distance migrants. A further assumption of the logistic fit specifically is that there is symmetry in the rate of acceleration and deceleration of occupancy about the inflection point. We also re-fit asymmetric Gompertz curves and found a tight relationship between the arrival dates estimated between the two methods (R^2^ = 0.95), with no systematic differences with latitude (*p* = 0.97). Although the suitability of a logistic curve to temporal occupancy patterns varies from time series to time series, the use of a consistently applied approximation is necessary for making comparison across time, space, and species.

We calculated MAD for every species-year-block combination in which the species was detected on at least 30 days during the arrival period (Julian days 80 to 180). We included only the species-year-block combinations in which the *r* ^2^ of the arrival date fit was at least 0.1 and in which the confidence interval was 40 days or less. For each species-block combination with at least five years of arrival date data, we conducted two simple linear regressions to explain variation in MAD. First, we modeled MAD as a function of year to provide estimates of the rate of change in arrival date through time as has been done in a number of studies (e.g., [17], [26]). Second, we modeled MAD as a function of minimum spring temperatures which may be more directly related to environmental cues affecting the timing of migration. Estimates of MAD were weighted inversely by the width of their confidence intervals in these analyses.

We calculated the median shifts in MAD in response to temperature for each species across all of the regions where sufficient data for that species was available. We then examined the extent to which interspecific variation in median phenological response could be explained by the our suite of species-specific variables: (1) migration distance (short vs. long), (2) foraging guild, (3) niche breadth, (4) population trend, and (5) relative abundance. A sixth derived variable, the average time for the migration front of a species to advance northward by 1° latitude, was also evaluated as a potential predictor. This was calculated as the slope of a regression of arrival day versus latitude. We examined models encompassing all possible combinations of these variables (*n* = 63), and model performance was assessed using the small sample–adjusted Akaike Information Criterion (AIC_c_; [48]). In addition, we calculated model weights (*w*) which reflect the weight of evidence in support of each model, and variable relative importance weights (*w*_+_) which are equal to the sum of all *w* over models in which the focal variable is a predictor [48].

## Supporting Information

Figure S1 **Regional temperature trends.** Trends of mean minimum spring temperature from 2000–2010 across 2°×2° lat-long blocks in eastern North America estimated from linear regression.
(TIF)

Appendix S1 **Arrival date maps.** For each of the 18 species examined in this study, estimated arrival dates are mapped for those lat-long blocks meeting data quality standards for each year from 2002–2010.
(PDF)
