# Supplementary figures and images for: Spatiotemporal Variation in Avian Migration Phenology: Citizen Science Reveals Effects of Climate Change
Source: PLoS One. 2012 Feb 22;7(2):e31662. doi: 10.1371/journal.pone.0031662 (PMC3285173; doi:10.1371/journal.pone.0031662)

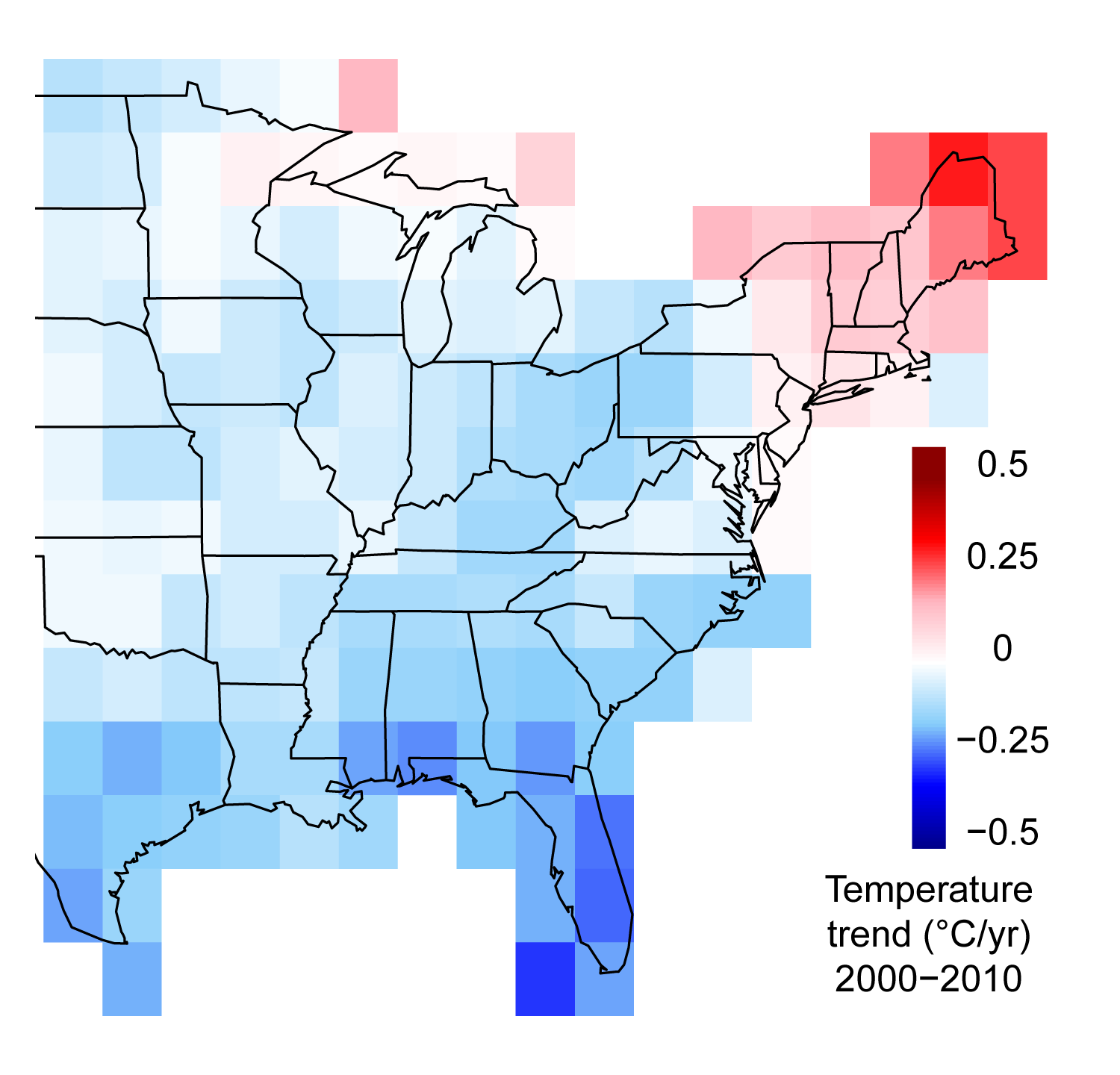

Supplement: Figure S1 — Regional temperature trends. Trends of mean minimum spring temperature from 2000–2010 across 2°×2° lat-long blocks in eastern North America estimated from linear regression. (TIF) [file pone.0031662.s001.tif]
