# Supplementary material for: Spatiotemporal Variation in Avian Migration Phenology: Citizen Science Reveals Effects of Climate Change
Source: PLoS One. 2012 Feb 22;7(2):e31662. doi: 10.1371/journal.pone.0031662 (PMC3285173; doi:10.1371/journal.pone.0031662)

Geothlypis trichas

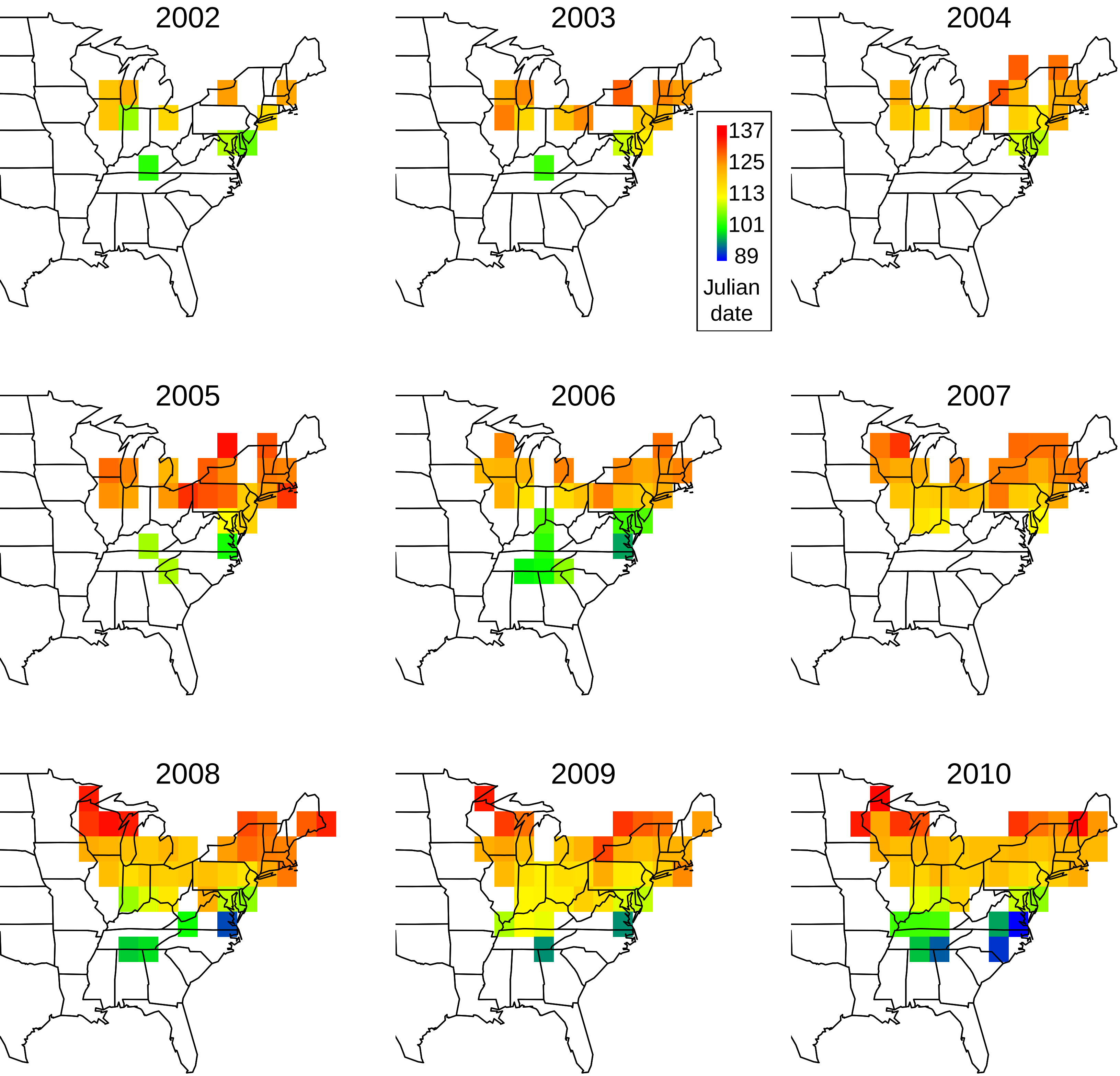

Icterus galbula

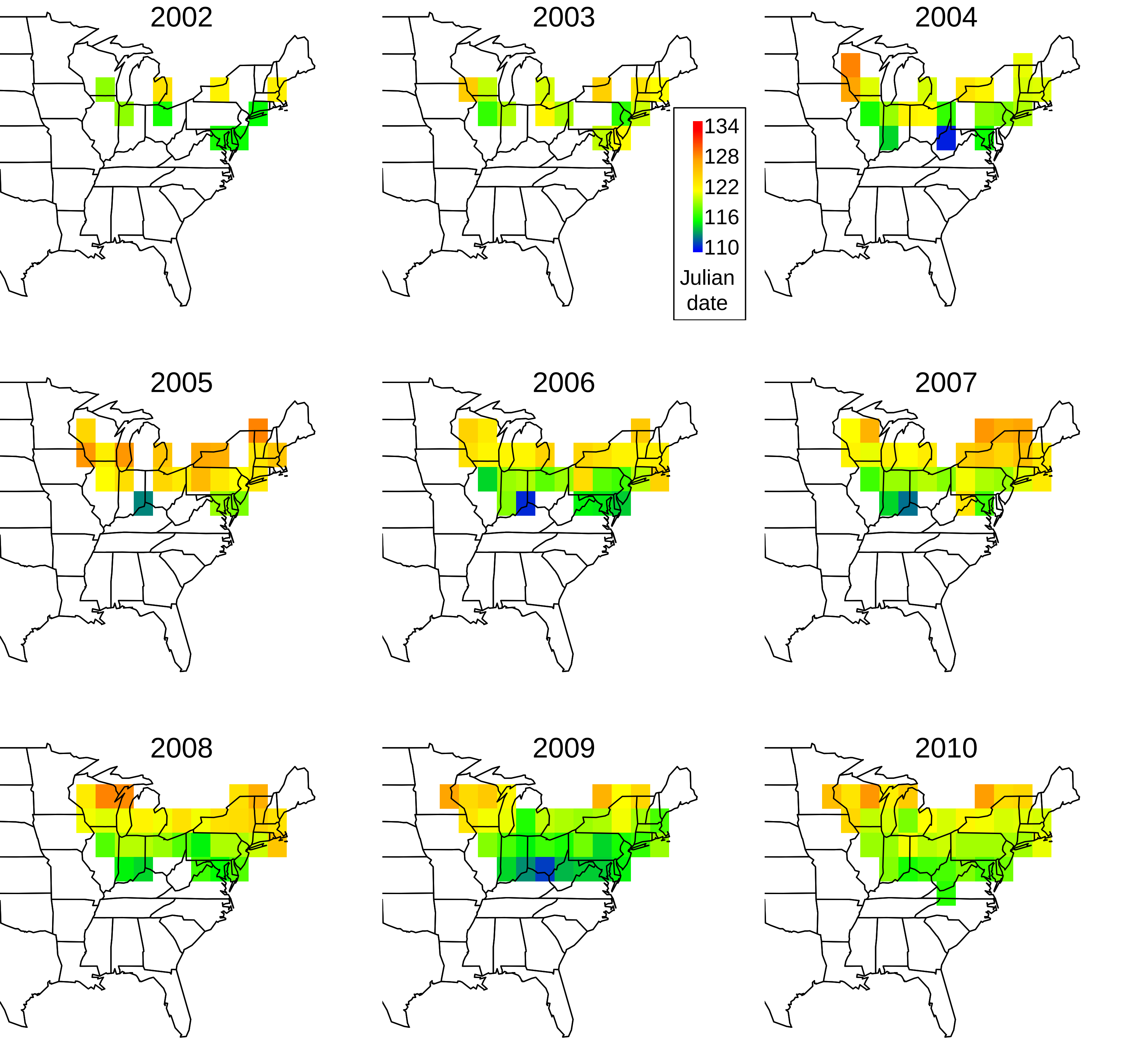

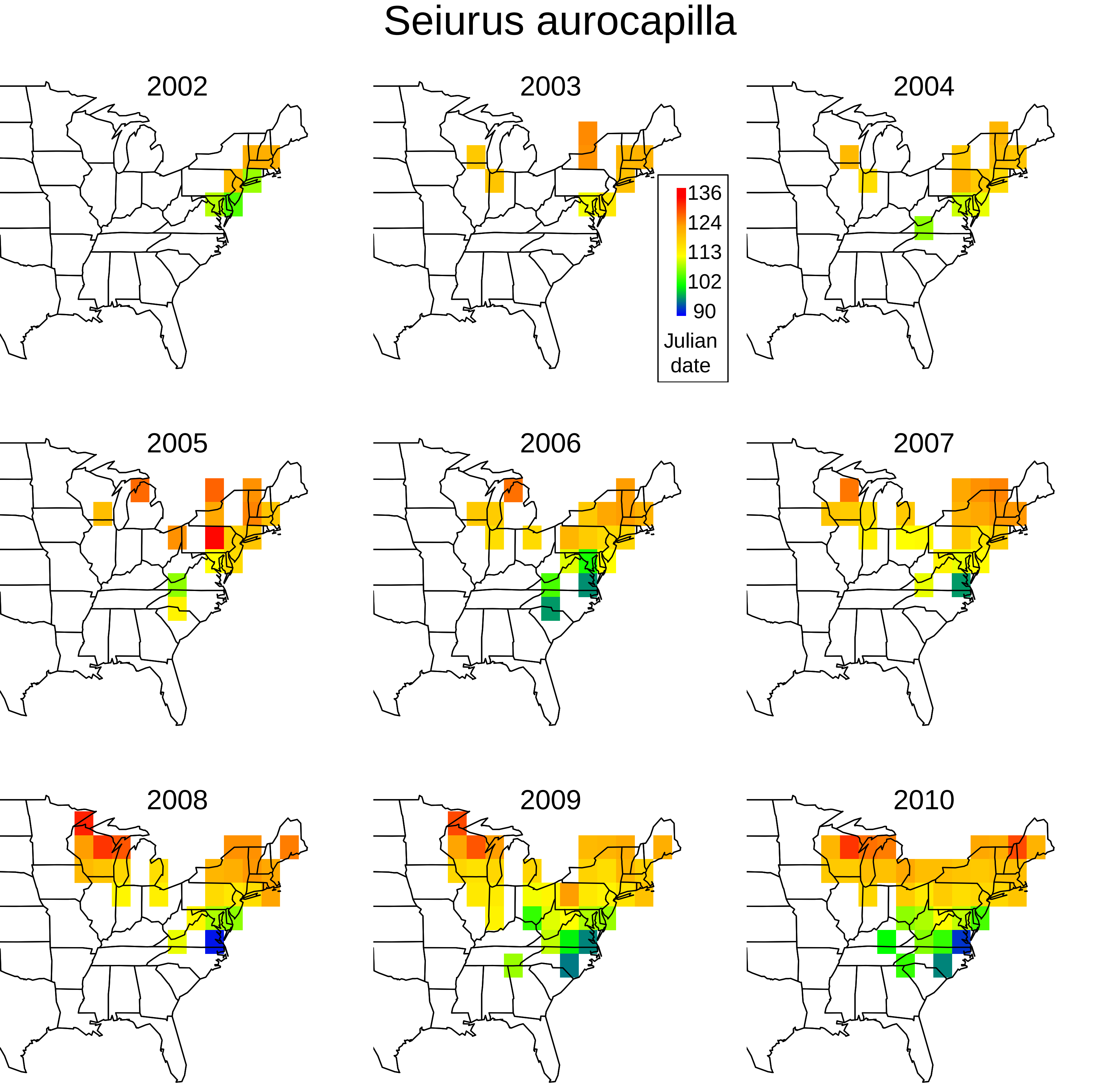

Seiurus aurocapilla
2002
2003
2004
2005
2006
2007
2008
2009
2010
136
124
113
102
90
Julian
date

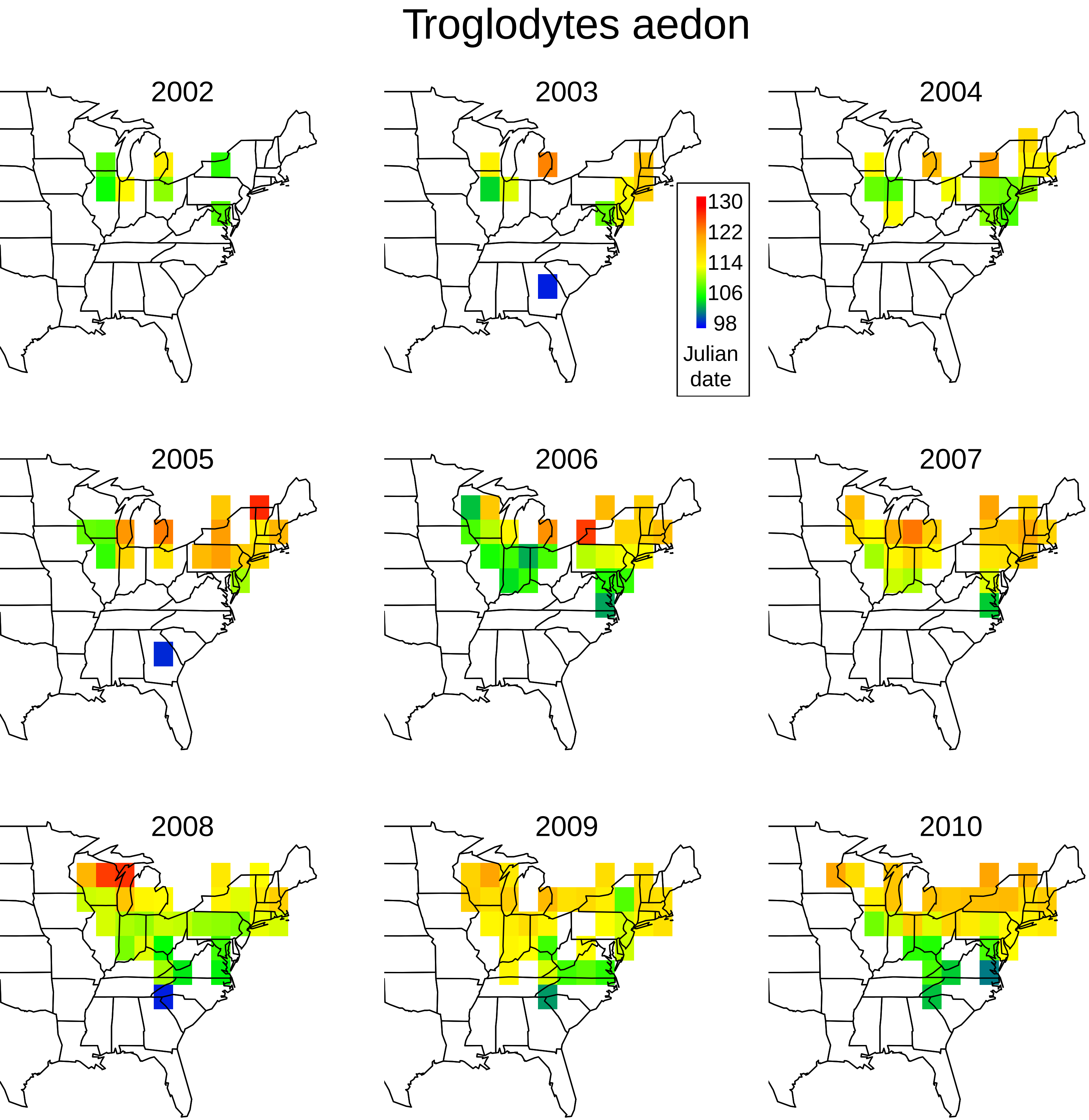
Troglodytes aedon
2002
2003
2004
2005
2006
2007
2008
2009
2010
130
122
114
106
98
Julian date

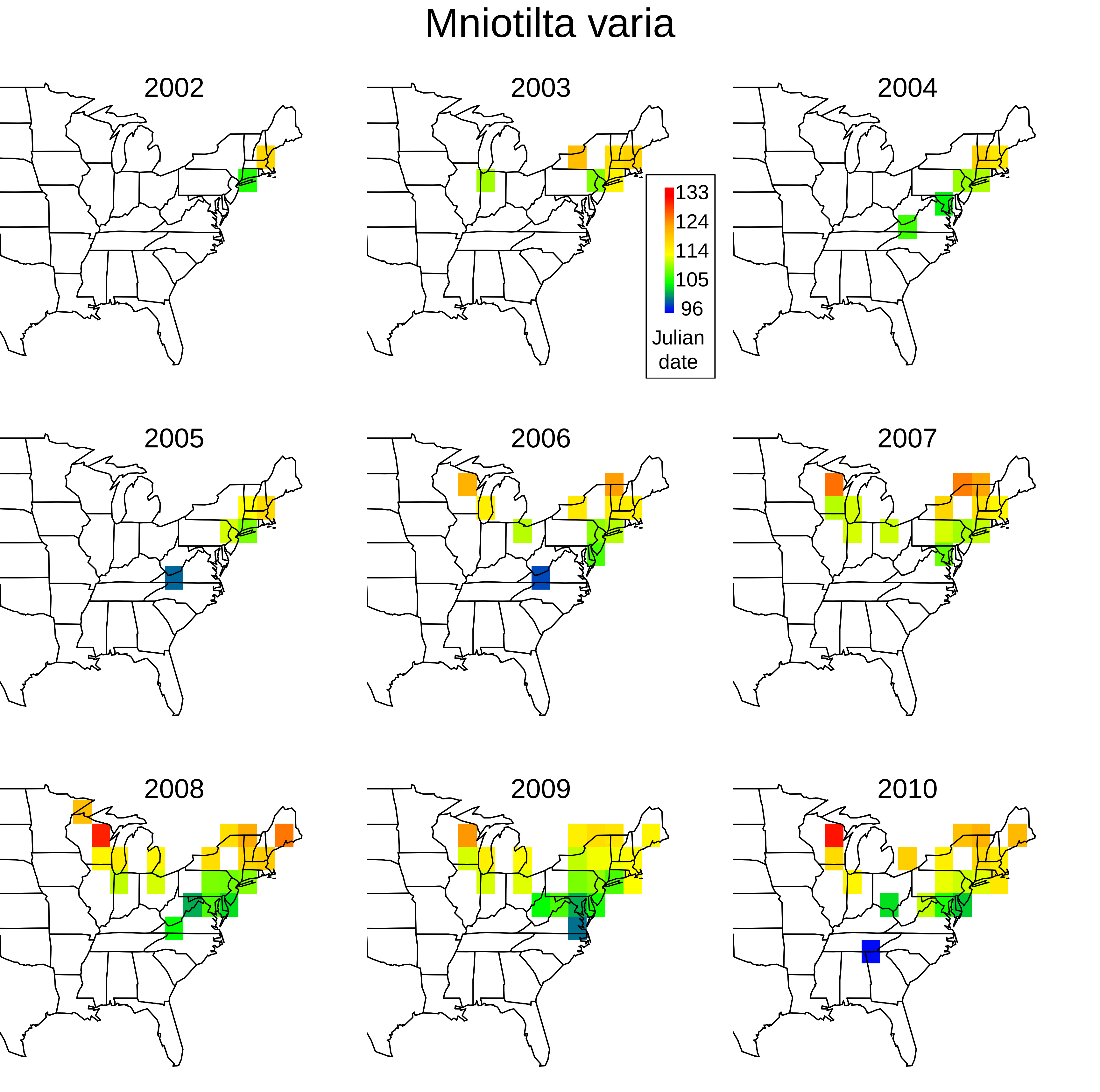
Mniotilta varia
2002
2003
2004
2005
2006
2007
2008
2009
2010
133
124
114
105
96
Julian
date

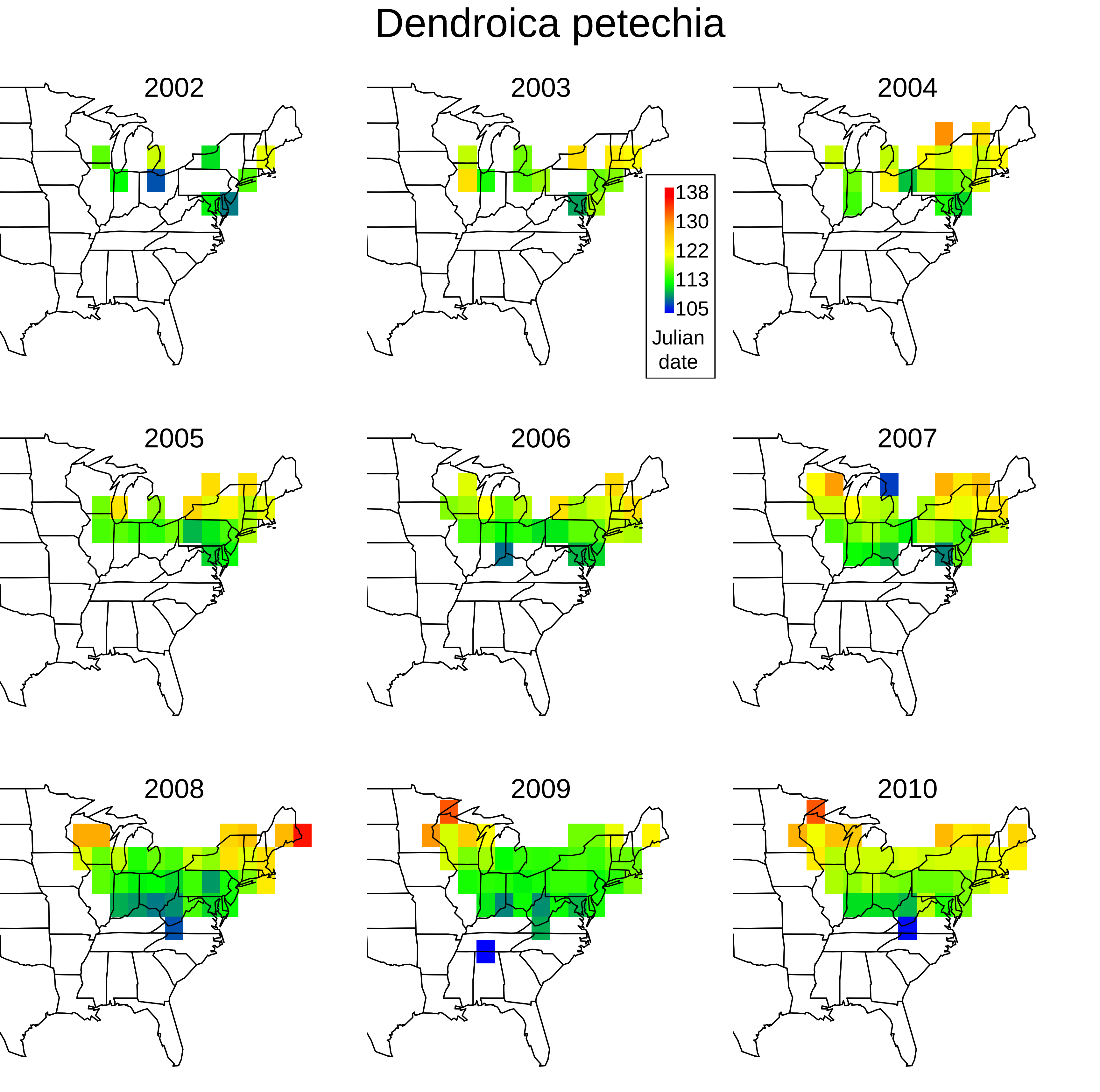

Dendroica petechia
2002
2003
2004
2005
2006
2007
2008
2009
2010
138
130
122
113
105
Julian date

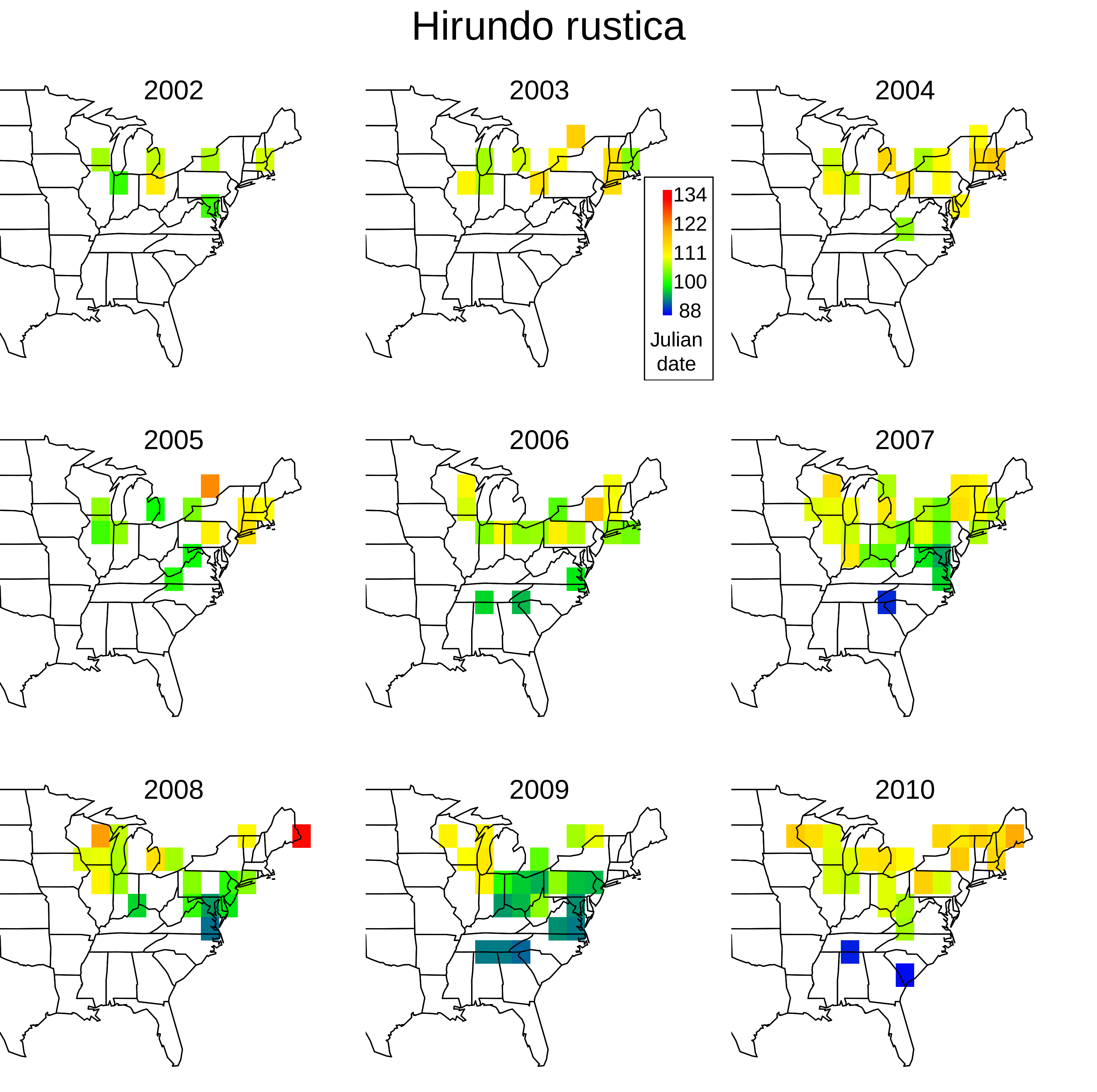
Hirundo rustica
2002
2003
2004
2005
2006
2007
2008
2009
2010
134
122
111
100
88
Julian
date

Hylocichla mustelina

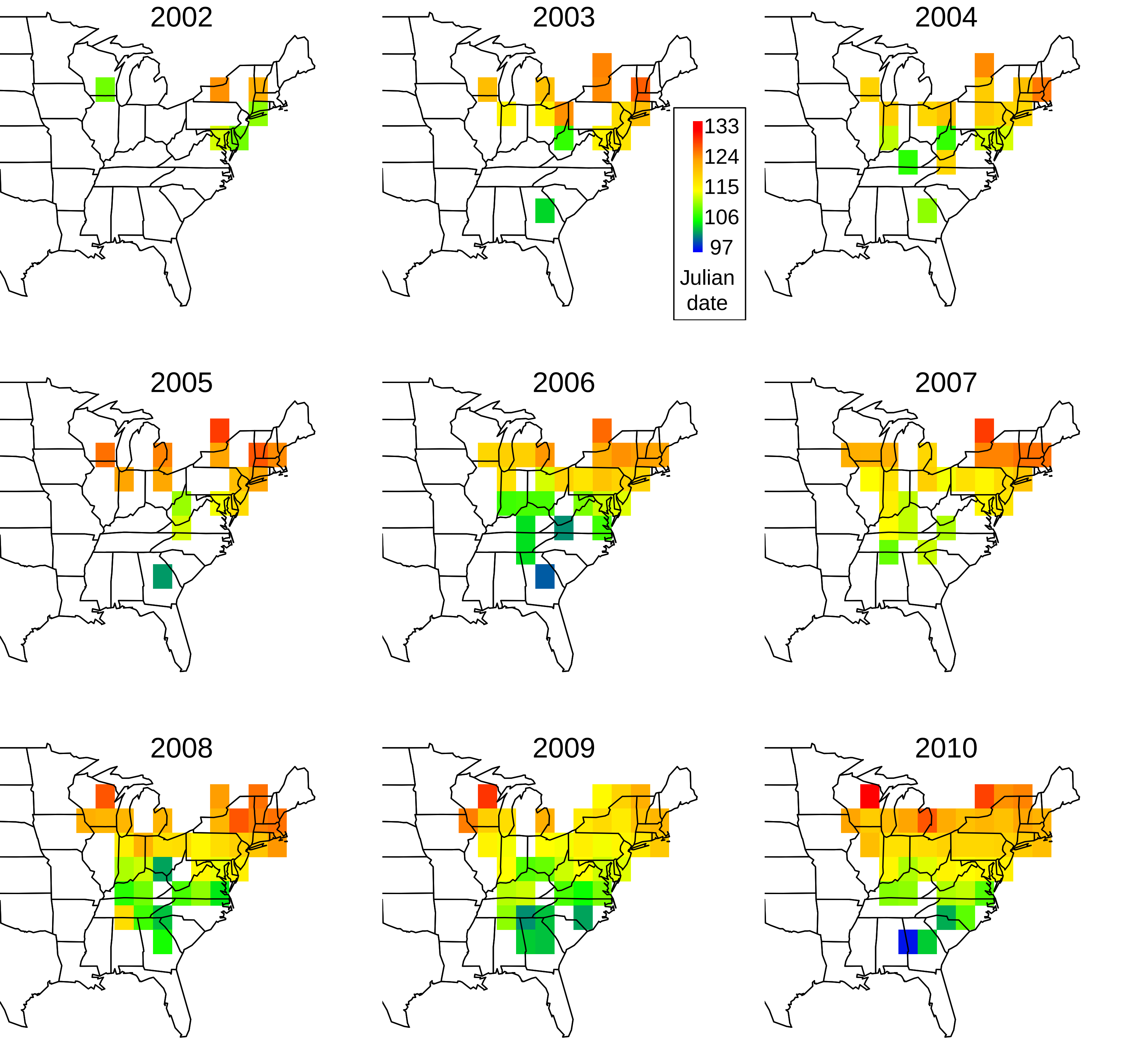

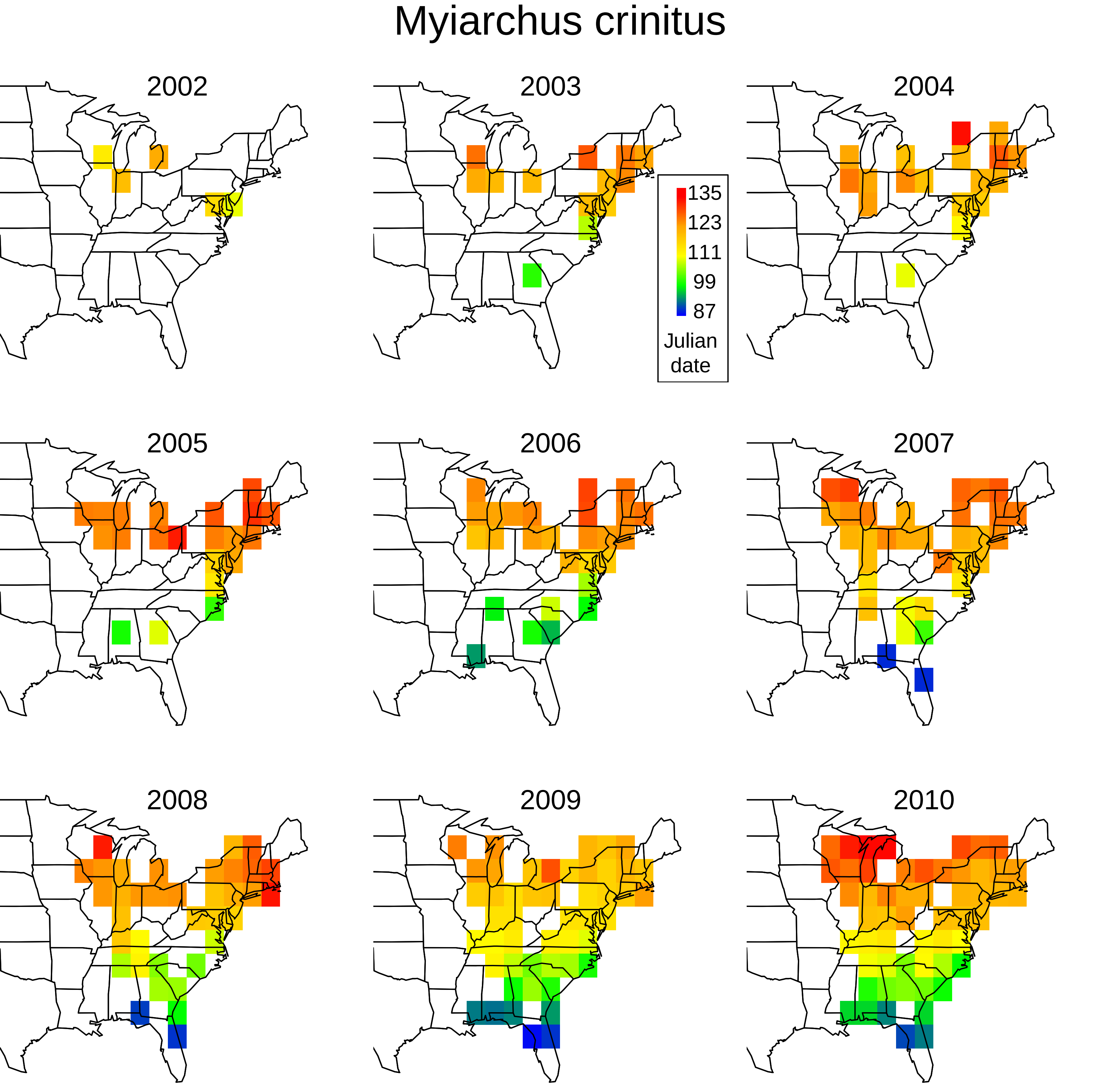
Myiarchus crinitus
2002
2003
2004
2005
2006
2007
2008
2009
2010
135
123
111
99
87
Julian
date

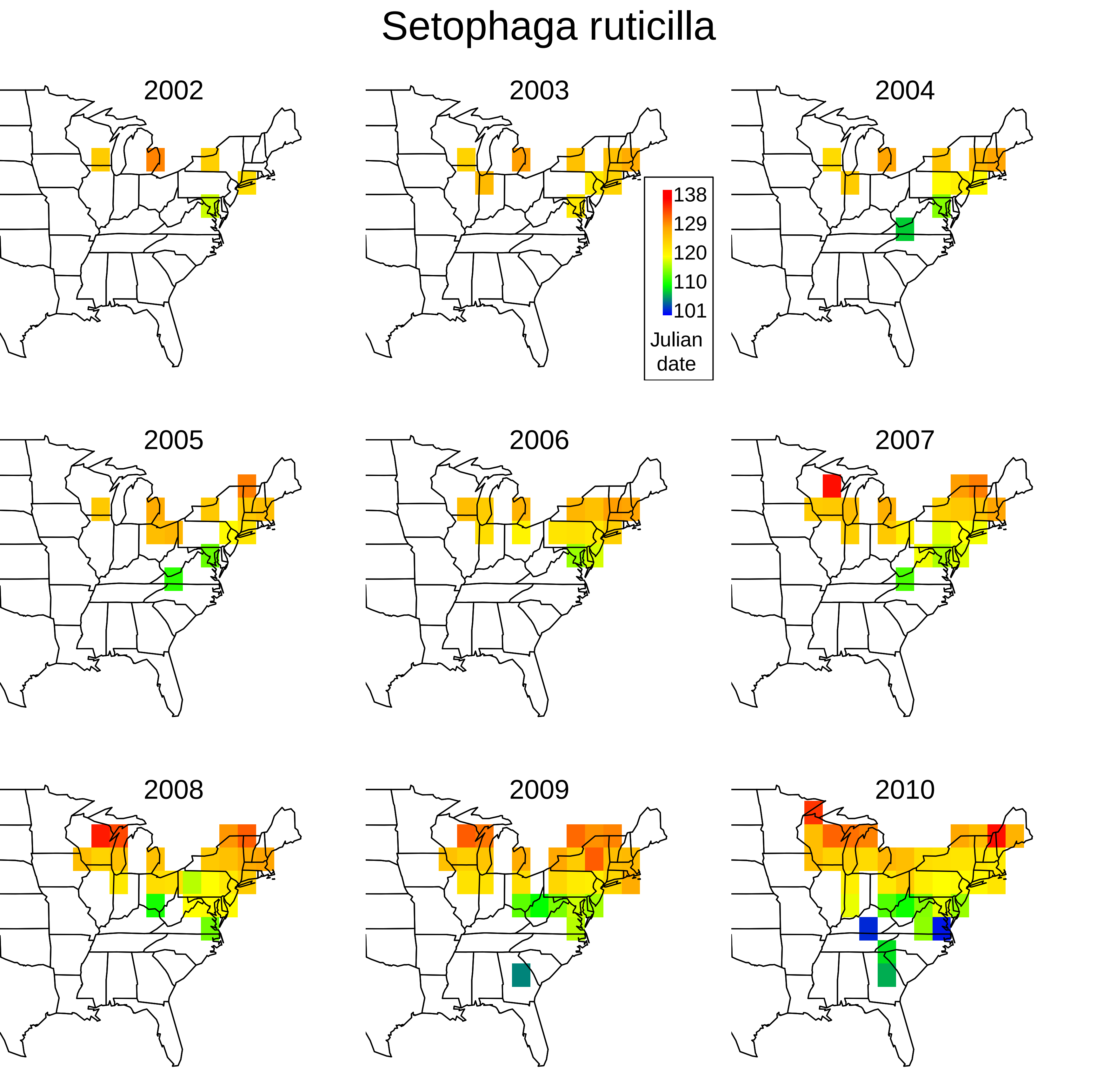
Setophaga ruticilla
2002
2003
2004
2005
2006
2007
2008
2009
2010
138
129
120
110
101
Julian date

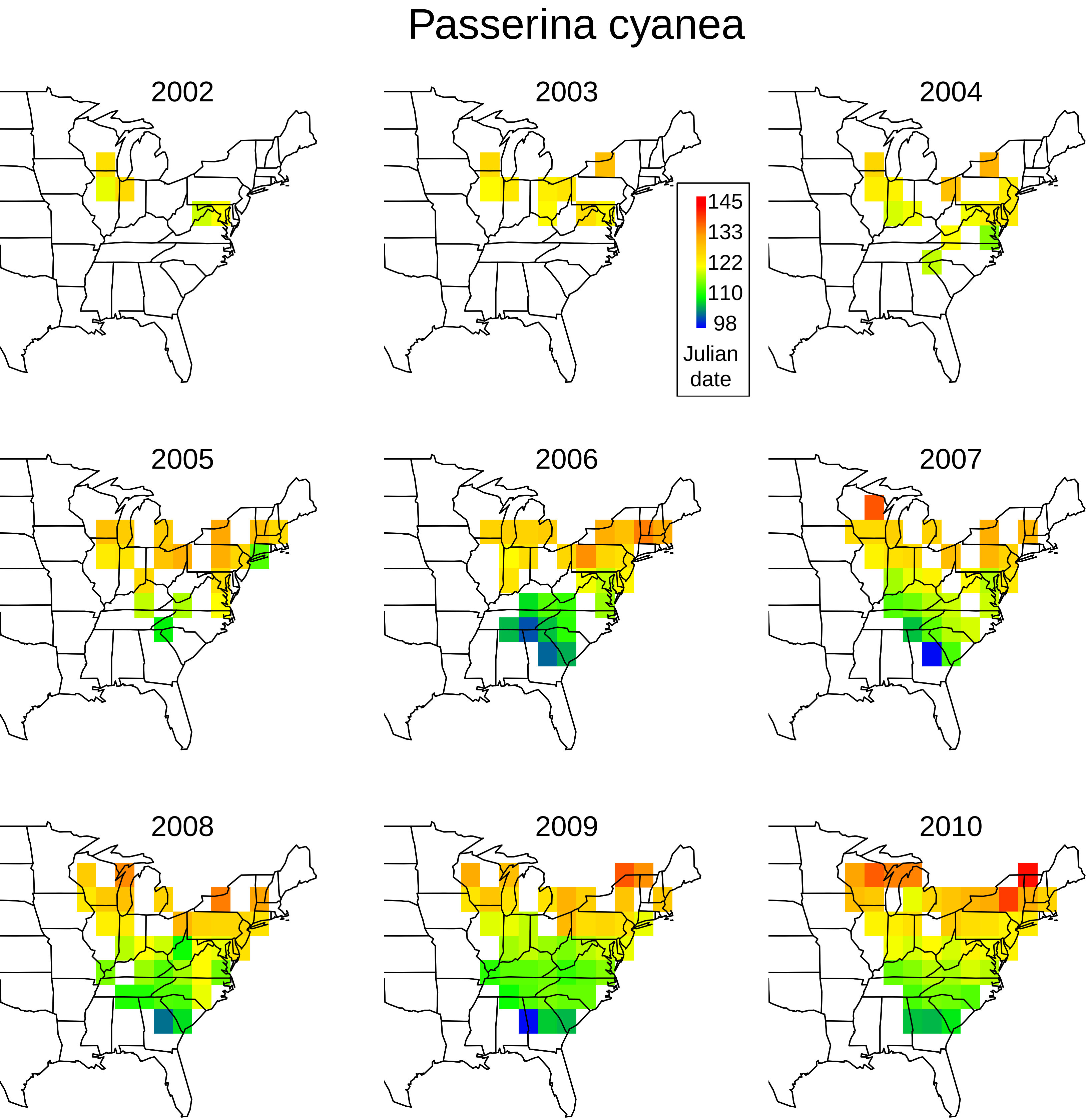
Passerina cyanea
2002
2003
2004
2005
2006
2007
2008
2009
2010
145
133
122
110
98
Julian date

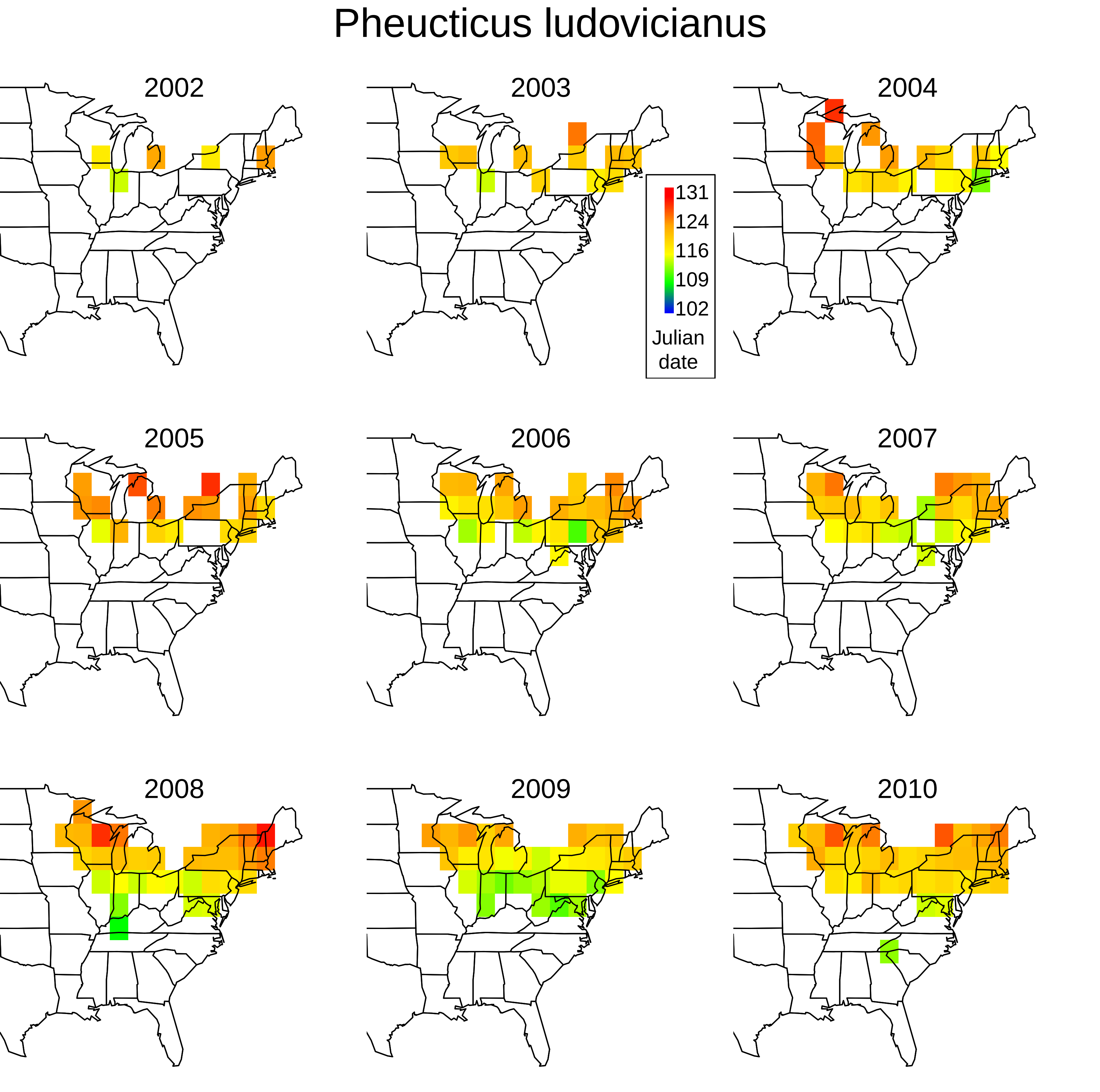
Pheucticus ludovicianus
2002
2003
2004
2005
2006
2007
2008
2009
2010
131
124
116
109
102
Julian
date

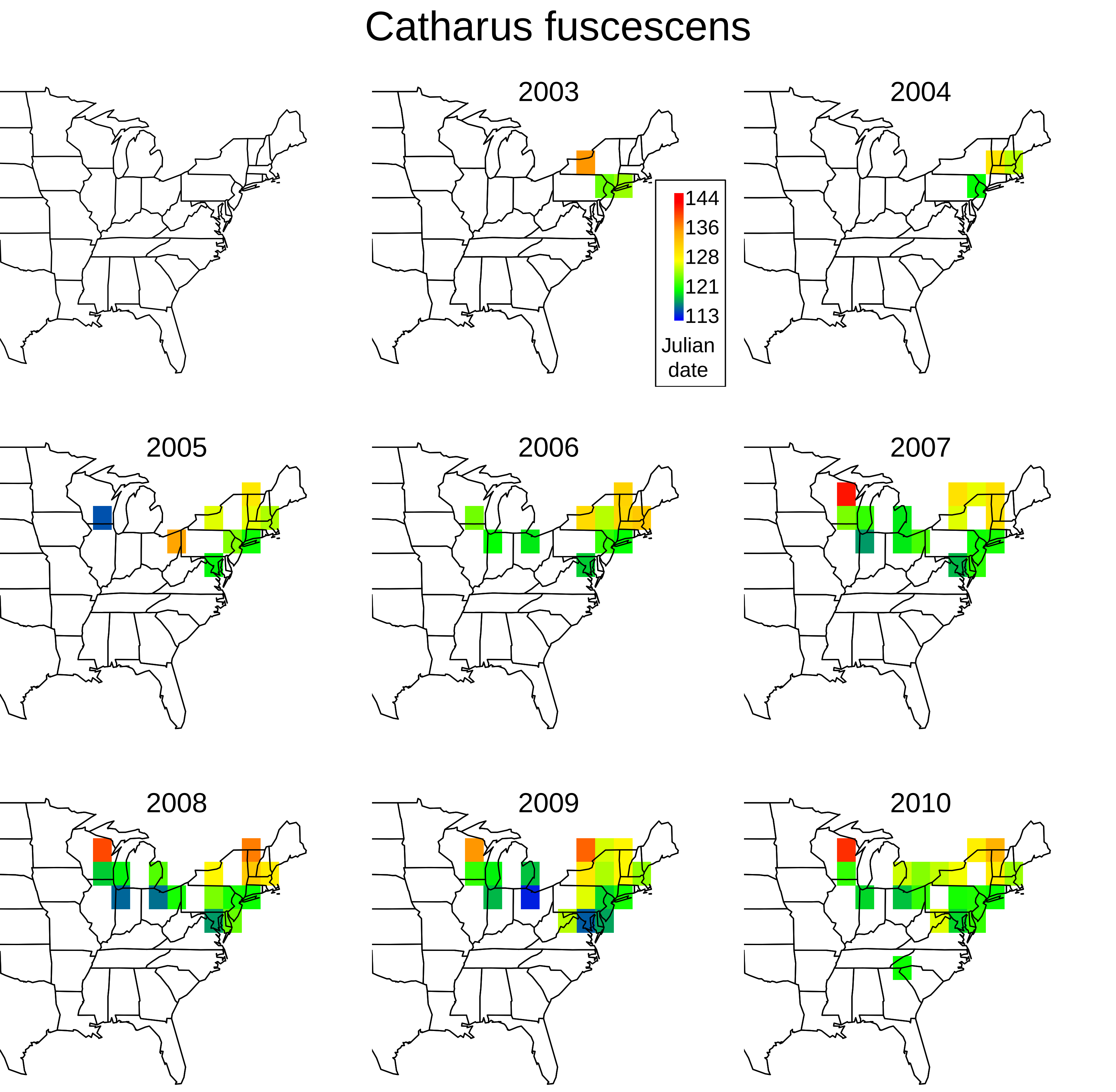

Catharus fuscescens
2003
2004
2005
2006
2007
2008
2009
2010
144
136
128
121
113
Julian date

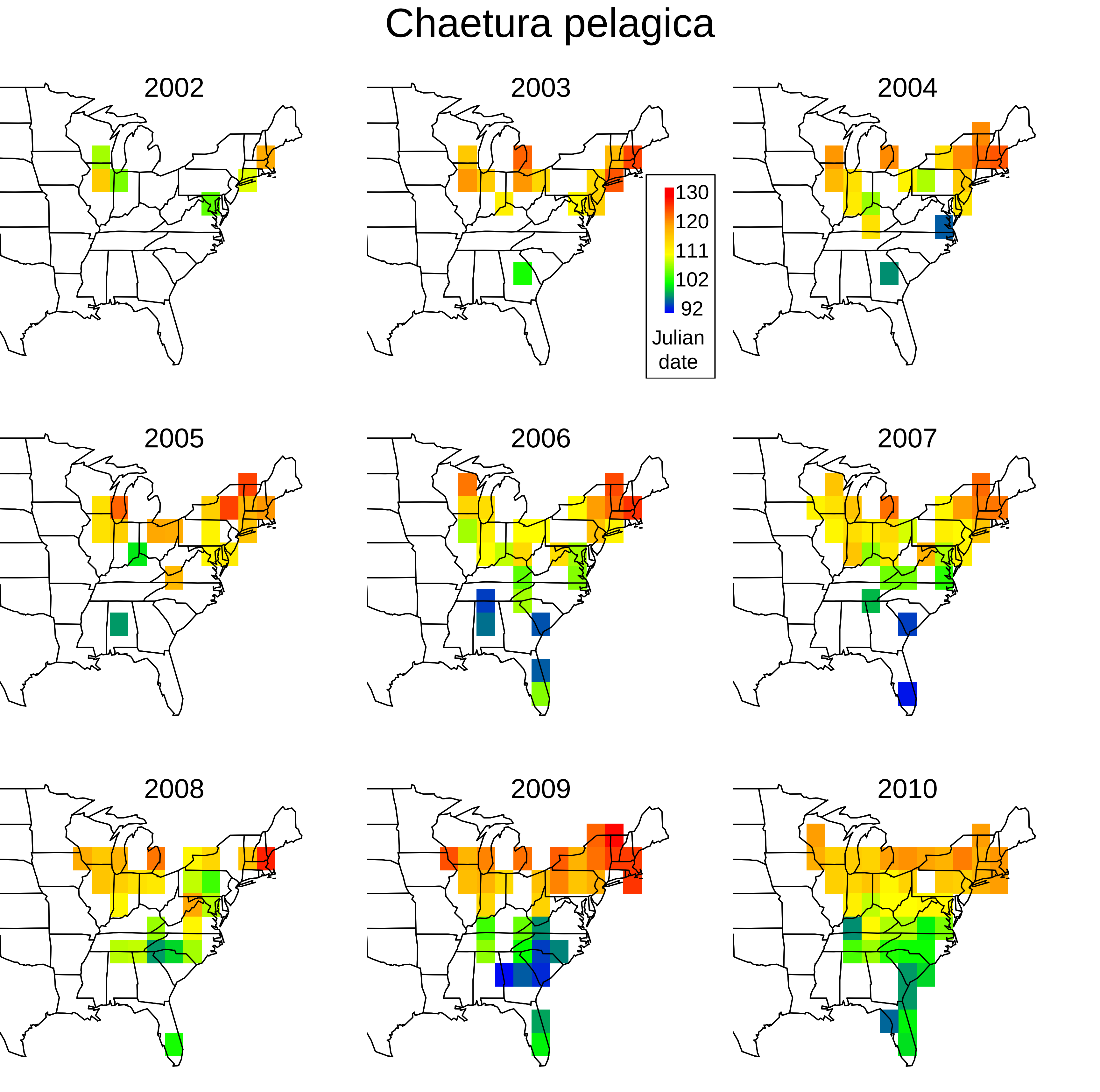
Chaetura pelagica
2002
2003
2004
2005
2006
2007
2008
2009
2010
130
120
111
102
92
Julian
date

Vireo olivaceus

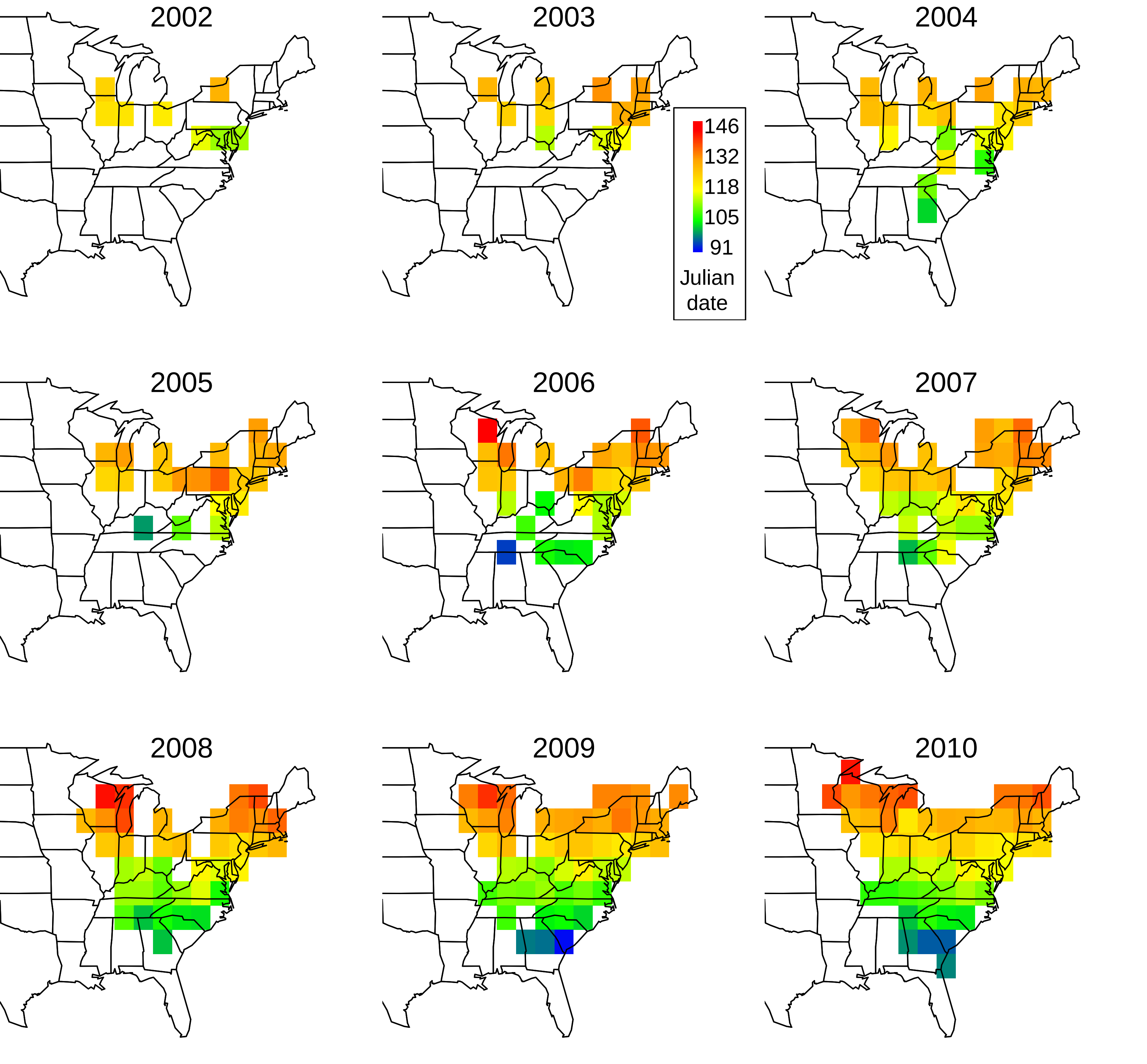

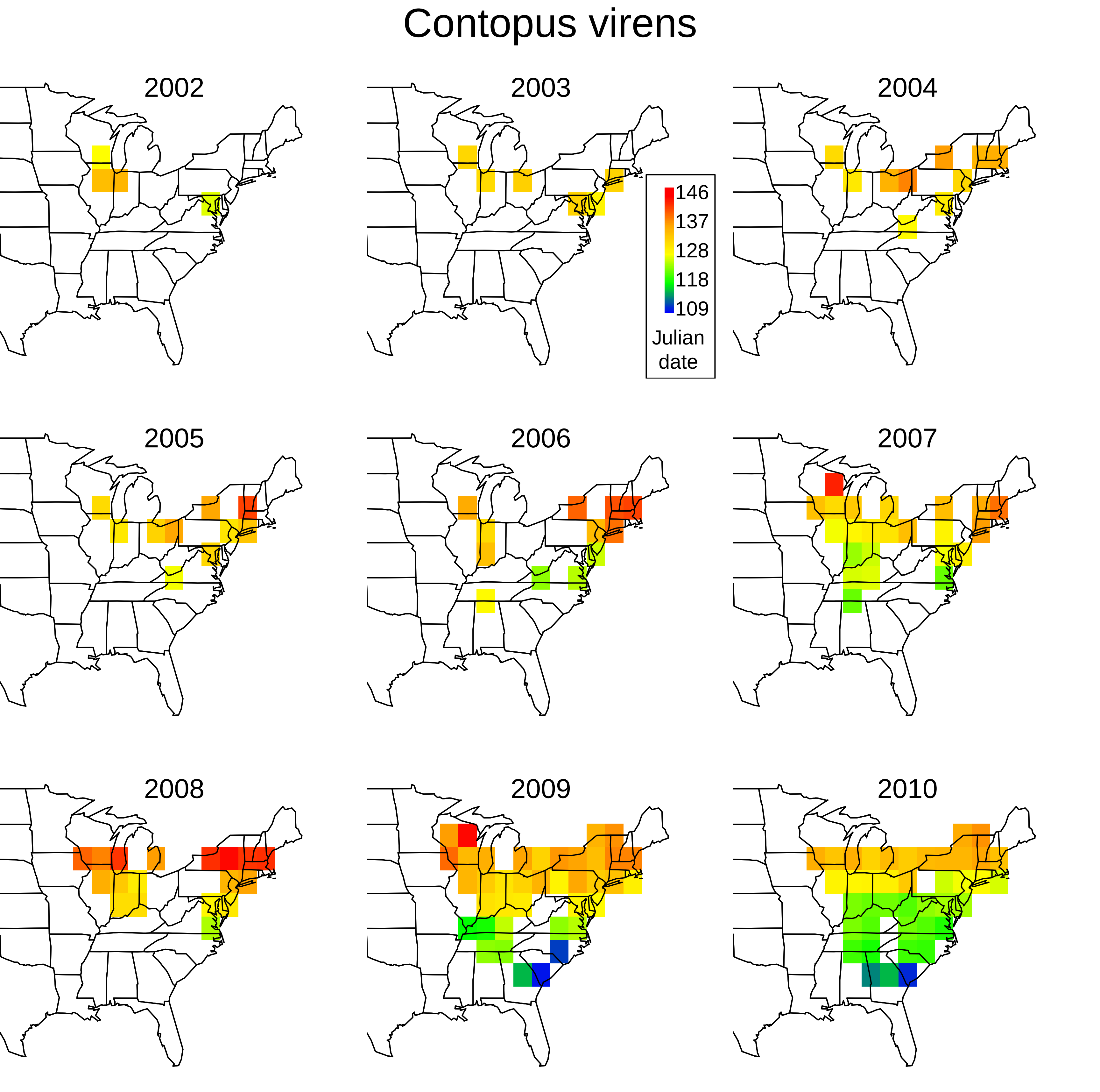
Contopus virens
2002
2003
2004
2005
2006
2007
2008
2009
2010
146
137
128
118
109
Julian
date

Piranga olivacea

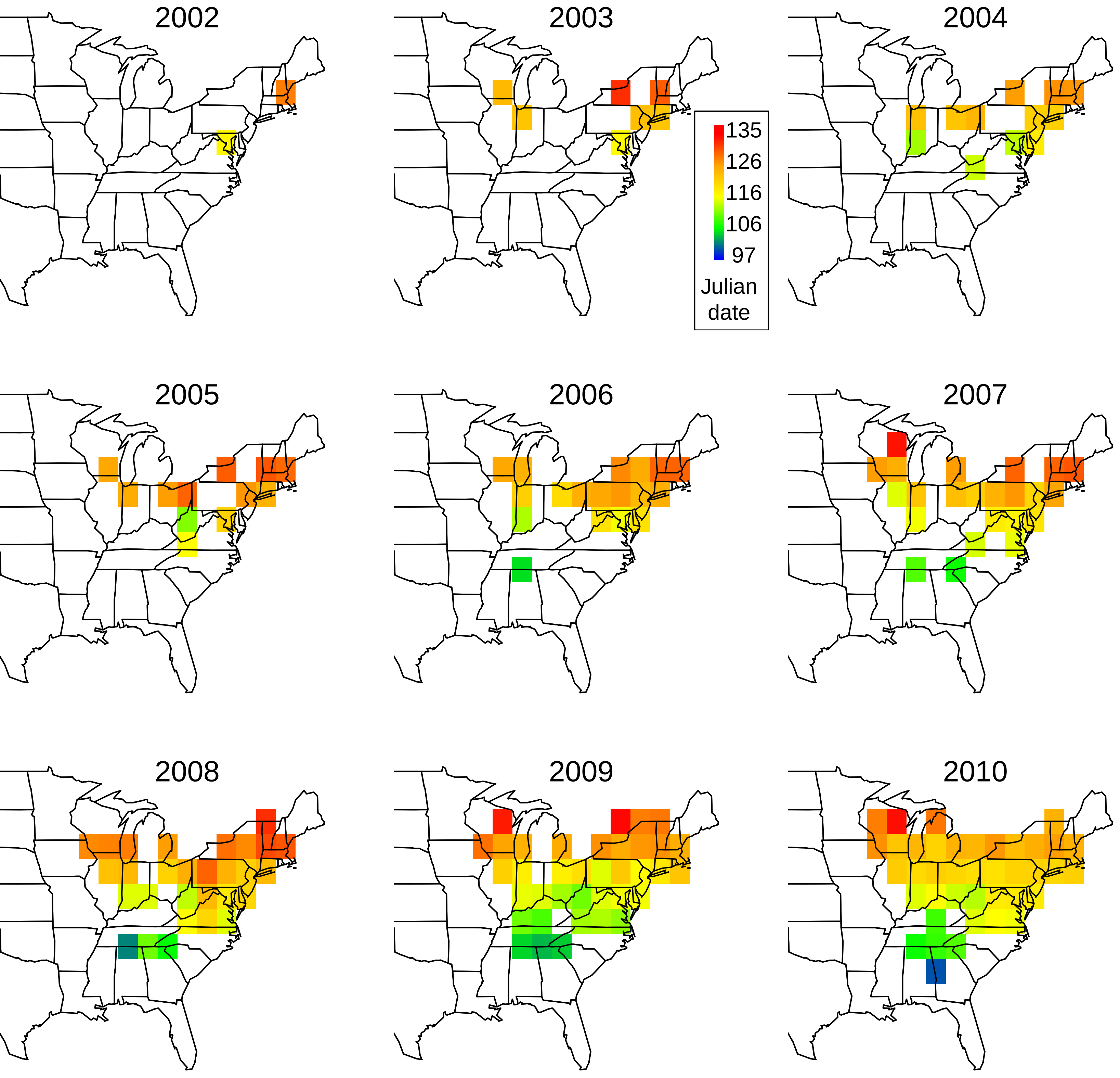

Tyrannus tyrannus

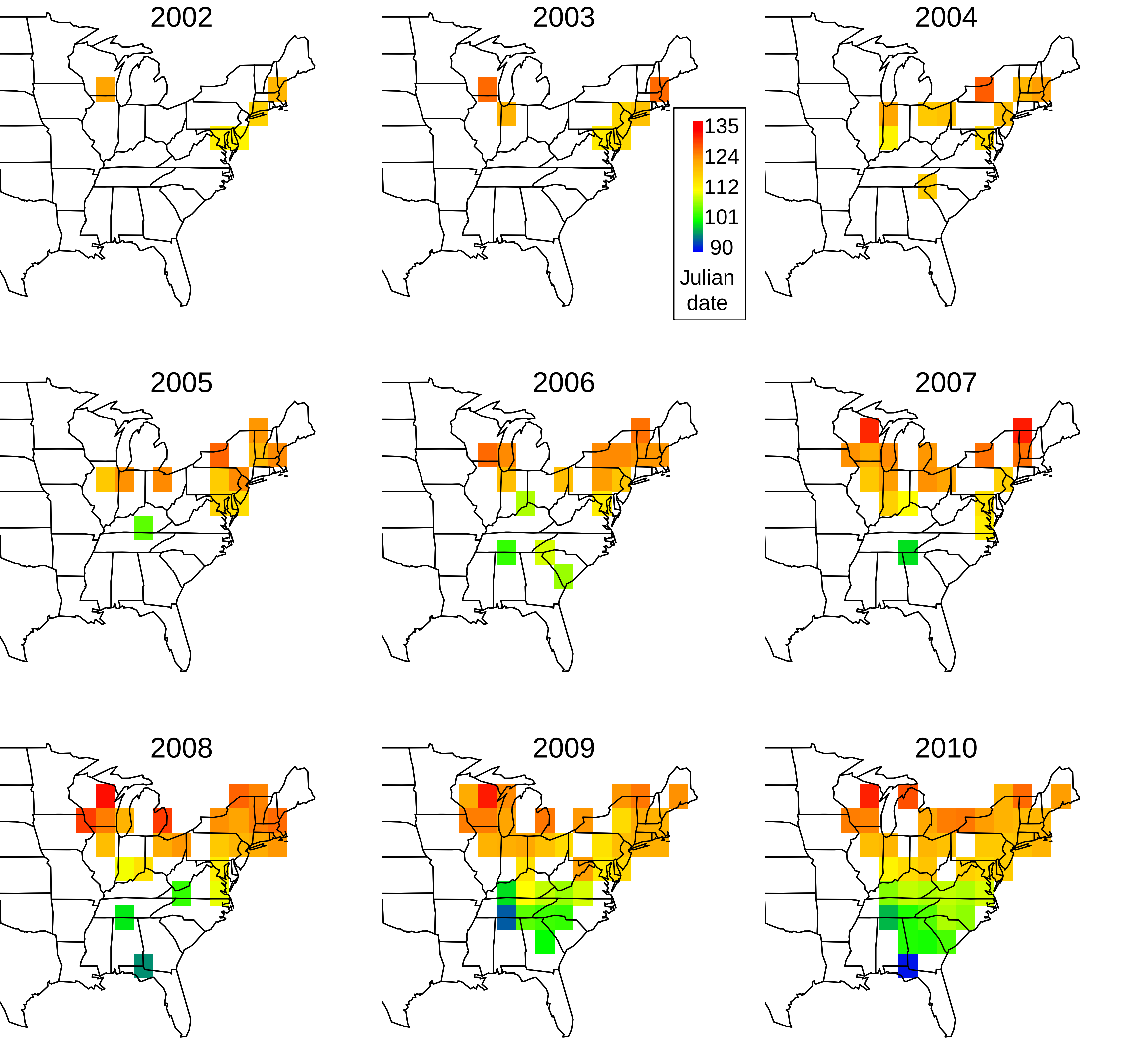

Supplement: Appendix S1 — Arrival date maps. For each of the 18 species examined in this study, estimated arrival dates are mapped for those lat-long blocks meeting data quality standards for each year from 2002–2010. (PDF) [file pone.0031662.s002.pdf]
